# Distinct pro-inflammatory properties of myeloid cell–derived apolipoprotein E2 and E4 in atherosclerosis promotion

**DOI:** 10.1016/j.jbc.2021.101106

**Published:** 2021-08-21

**Authors:** Emily Igel, April Haller, Patrick R. Wolfkiel, Melissa Orr-Asman, Anja Jaeschke, David Y. Hui

**Affiliations:** Department of Pathology and Laboratory Medicine, Metabolic Diseases Research Center, University of Cincinnati College of Medicine, Cincinnati, Ohio, USA

**Keywords:** ApoE polymorphism, inflammation, inflammasome, oxidative stress, cholesterol efflux, atherosclerosis, bone marrow transplant, ApoE, apolipoprotein E, BSA, bovine serum albumin, HDL, high-density lipoproteins, HSC, hematopoietic stem cells, IL, interleukin, LDL, low-density lipoproteins, LPS, lipopolysaccharides, NLRP3, NOD-, LRR-, and pyrin domain-containing protein 3, oxLDL, oxidized LDL, TNFα, tumor necrosis factor α

## Abstract

Polymorphisms in the apolipoprotein E (apoE) gene are risk factors for chronic inflammatory diseases including atherosclerosis. The gene product apoE is synthesized in many cell types and has both lipid transport–dependent and lipid transport–independent functions. Previous studies have shown that apoE expression in myeloid cells protects against atherogenesis in hypercholesterolemic *ApoE*^*−/−*^ mice. However, the mechanism of this protection is still unclear. Using human *APOE* gene replacement mice as models, this study showed that apoE2 and apoE4 expressed endogenously in myeloid cells enhanced the inflammatory response *via* mechanisms independent of plasma lipoprotein transport. The data revealed that apoE2-expressing myeloid cells contained higher intracellular cholesterol levels because of impaired efflux, causing increasing inflammasome activation and myelopoiesis. In contrast, intracellular cholesterol levels were not elevated in apoE4-expressing myeloid cells, and its proinflammatory property was found to be independent of inflammasome signaling and related to enhanced oxidative stress. When *ApoE*^*−/−*^ mice were reconstituted with bone marrow from various human *APOE* gene replacement mice, effective reduction of atherosclerosis was observed with marrow cells obtained from *APOE3* but not *APOE2* and *APOE4* gene replacement mice. Taken together, these results documented that apoE2 and apoE4 expression in myeloid cells promotes inflammation *via* distinct mechanisms and promotes atherosclerosis in a plasma lipoprotein transport–independent manner.

The *APOE* gene encoding the apolipoprotein E (apoE) protein is one of the most widely studied genes in biomedical research (1). Its popularity owes in large part to the suitability of *ApoE*^*−/−*^ mice as an experimental animal model to study the pathogenesis of numerous diseases, as well as the association between *APOE* polymorphism and a wide spectrum of diseases in humans including coronary and peripheral vascular disease (2, 3, 4, 5, 6, 7, 8, 9, 10, 11, 12, 13), obesity/diabetes (10, 14, 15), and Alzheimer’s disease (16). The human *APOE* gene exists with three major polymorphic alleles, ε2, ε3, and ε4, encoding apoE2, apoE3, and apoE4, respectively. The ε3 allele is the most common allele with an allelic frequency of ∼75%, while ∼15% of the population are carriers of the ε4 allele and ∼8% of the population are carriers of the ε2 allele. The ε4 allele is associated with an ∼1.7-fold increased risk of cardiovascular diseases (2, 17, 18, 19), whereas carriers of the ε2 allele typically have normal or reduced plasma cholesterol levels but have higher plasma triglyceride levels (20, 21) and are prone to develop type III dysbetalipoproteinemia (17). The ε2 allele increases the risk and severity of coronary artery disease in diabetes (10, 12, 15, 21, 22) and is also an independent risk factor for peripheral vascular disease and carotid artery atherosclerosis in nondiabetics (3, 4, 5). Interestingly, whereas the ε4 allele is associated with an increased risk and lower age of onset of Alzheimer’s disease, the ε2 allele appears to be protective against Alzheimer’s disease pathogenesis (23).

ApoE is synthesized in many cell types including hepatocytes, adipocytes, macrophages, smooth muscle cells, and in the brain (24, 25, 26). It has multiple functions both intracellularly and extracellularly in mediating lipid transport and cell homeostasis (24, 25, 26). ApoE synthesized in hepatocytes is secreted into the plasma circulation, where it serves as the ligand that mediates lipoprotein binding to low-density lipoprotein (LDL) receptor family proteins and cell surface heparan sulfate proteoglycans for the clearance of triglyceride-rich lipoproteins from the plasma circulation (27, 28). The interaction between extracellular apoE and specific cell surface receptors in the LDL receptor–related protein family also regulates cell signaling events in various cell types, including the activation of endothelial nitric oxide synthase in endothelial cells (29, 30), inhibition of vascular smooth muscle cell proliferation and migration (31), and suppression of toll-like receptor activation by increasing macrophage polarization toward the anti-inflammatory M2 phenotype (32).

Dysfunctional apoE with impairment in lipoprotein transport and/or cell signal regulatory functions increases the risk of disease pathogenesis. For example, apoE2 is defective in binding to the LDL receptor (33, 34). Hence, ε2 carriers are prone to develop dysbetalipoproteinemia with an increased risk of atherosclerosis (35). In contrast, apoE4 binds LDL receptor family proteins with high affinity, but it fails to activate endothelial nitric oxide synthase and impedes endothelial repair after vascular injury (30). The impairment in endothelial nitric oxide synthase activation may be one mechanism underlying the increased risk of both cardiovascular and neurodegenerative diseases in ε4 carriers (36).

ApoE is also synthesized in cells of the myeloid lineage, where its dysfunction may also contribute to cardiovascular and neurodegenerative diseases. In particular, apoE expressed in macrophages has been shown to be necessary, and systemic apoE is not sufficient to mobilize intracellular cholesterol for efflux from macrophage foam cells in the initial step of the reverse cholesterol transport pathway (37). The impairment of cholesterol efflux with apoE deficiency not only leads to foam cell formation but also promotes myelopoiesis to exacerbate inflammatory response (38). The importance of macrophage-derived apoE in protection from atherosclerosis is documented in bone marrow transplantation studies showing that the reconstitution of macrophage apoE expression was sufficient to prevent atherosclerosis in hypercholesterolemic *ApoE*^*−/−*^ mice (39, 40, 41), whereas the transplantation of apoE-deficient bone marrow was found to exacerbate atherosclerosis in *Ldlr*^*−/−*^ and C57BL/6 WT mice with physiologically regulated apoE expression in the liver (42, 43). Interestingly, *in vitro* studies using mouse J774A.1 macrophages expressing various apoE isoforms revealed isoform-specific differences in pro-inflammatory cytokine secretion in response to lipopolysaccharide (LPS) stimulation (44). However, the underlying mechanism for the isoform-specific differences and the potential impact on atherosclerosis have not been identified. The objective of this study is to delineate the mechanism by which different apoE isoforms expressed in myeloid cells influence inflammation and the consequential effect on atherosclerosis.

## Results

### ApoE2 and apoE4 enhanced pro-inflammatory cytokine production in LPS-stimulated leukocytes

**Figure 1:**
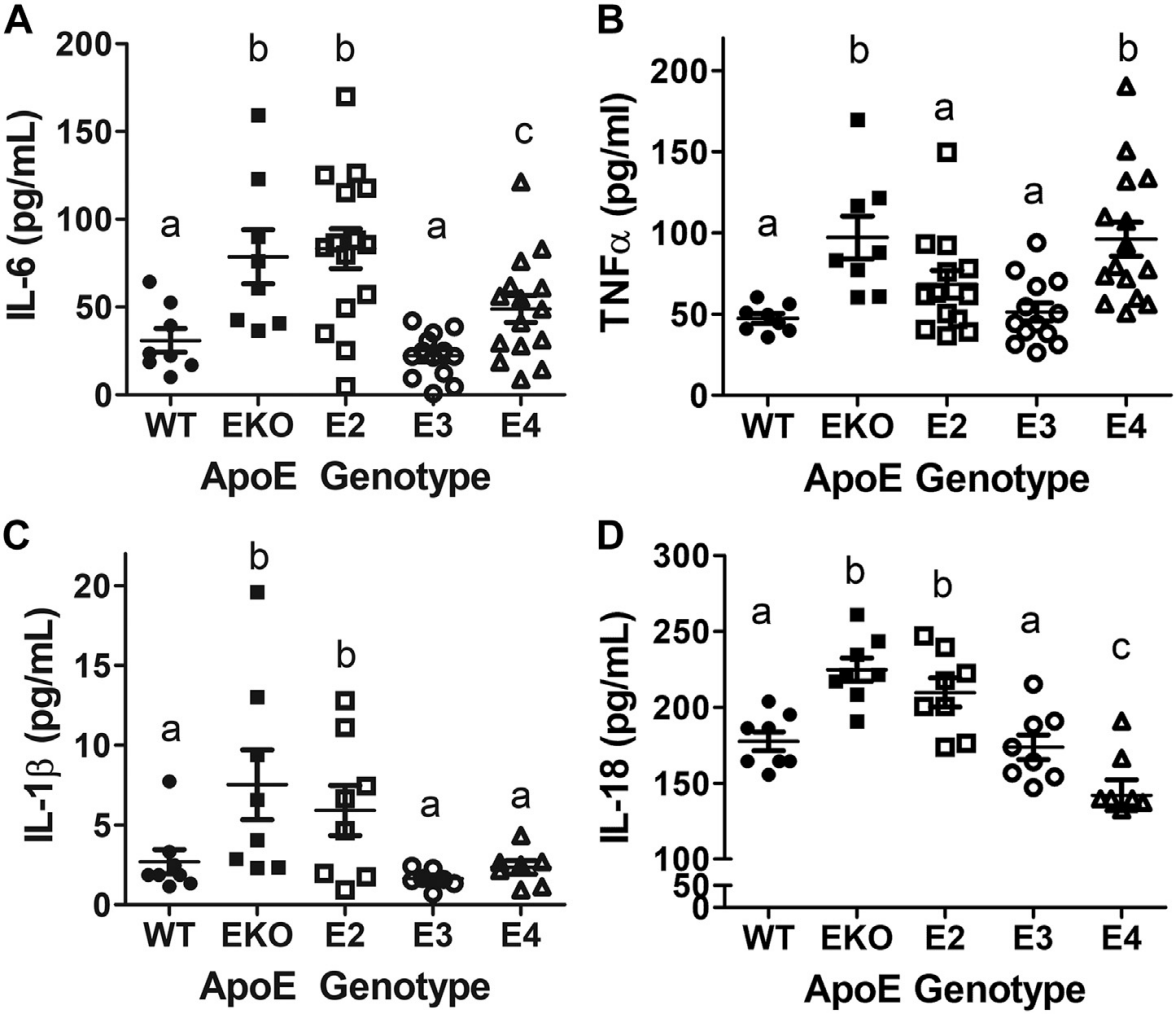
**ApoE2 and ApoE4 enhanced proinflammatory cytokine production in LPS-stimulated leukocytes.** Blood obtained from C57BL/6J WT and *ApoE*^*−/−*^ mice as well as human *APOE2*, *APOE3*, and *APOE4* gene replacement mice was incubated with 100 ng/ml LPS for 4 h. Plasma was obtained after incubation for ELISA determination of (*A*) IL-6, N = 15; (*B*) TNFα, N = 15; (*C*) IL-1β, N = 8; and (*D*) IL-18, N = 8. All data were evaluated by one-way ANOVA with Student–Newman–Keuls post hoc analysis for comparison between groups. Groups with *different letters* indicate significant differences at *p* < 0.05. apoE, apolipoprotein E; IL, interleukin; LPS, lipopolysaccharide; TNFα, tumor necrosis factor α.

The association between ε2 and ε4 alleles in the *APOE* gene with increased risk of atherosclerosis, a chronic disease of inflammation and hyperlipidemia, in a manner that is independent of plasma lipid levels (3, 5, 45), suggested that apoE2 and apoE4 expression may elevate inflammatory response directly. Therefore, we used a widely accepted procedure developed in the clinical setting to study physiologically relevant blood leukocyte cytokine release to compare the inflammatory response of human *APOE2*, *APOE3*, and *APOE4* gene replacement mice *ex vivo* (46). Blood samples from C57BL/6J mice, expressing mouse apoE, and *ApoE*^*−/−*^ mice with no apoE expression were used as controls. Results showed that blood cells in *APOE3* gene replacement mice were similar to those in WT mice with low inflammatory cytokine production in response to LPS challenge (Fig. 1, *A*–*D*). In contrast, enhanced interleukin (IL)-6 production was observed from leukocytes of *APOE2* and *APOE4* gene replacement mice similar to that observed with blood from *ApoE*^*−/−*^ mice (Fig. 1*A*). Interestingly, significantly more tumor necrosis factor α (TNFα) was produced by cells in *APOE4* mice, similar to that observed in *ApoE*^*−/−*^ mice, but TNFα production by blood cells from *APOE2* mice was similar to that observed with blood cells from WT and *APOE3* mice (Fig. 1*B*). In contrast, higher levels of IL-1β and IL-18 were produced by *ApoE*^*−/−*^ and *APOE2* cells, but the levels of these cytokines were lower in *APOE3* and *APOE4* cells similar to WT controls (Fig. 1, *C* and *D*). The selectivity by which apoE2 and apoE4 differentially enhanced proinflammatory cytokine production suggests that these two apoE isoforms may activate immune response through different mechanisms. ApoE expression in leukocytes is restricted to cells in the myeloid lineage, and apoE is absent in lymphocytes (29, 47). Therefore, the enhanced leukocyte inflammatory response observed with blood isolated from *APOE2* and *APOE4* gene replacement mice is likely due to enhanced myeloid cell inflammatory response.

### ApoE2 enhances inflammasome priming and activation in macrophages

**Figure 2:**
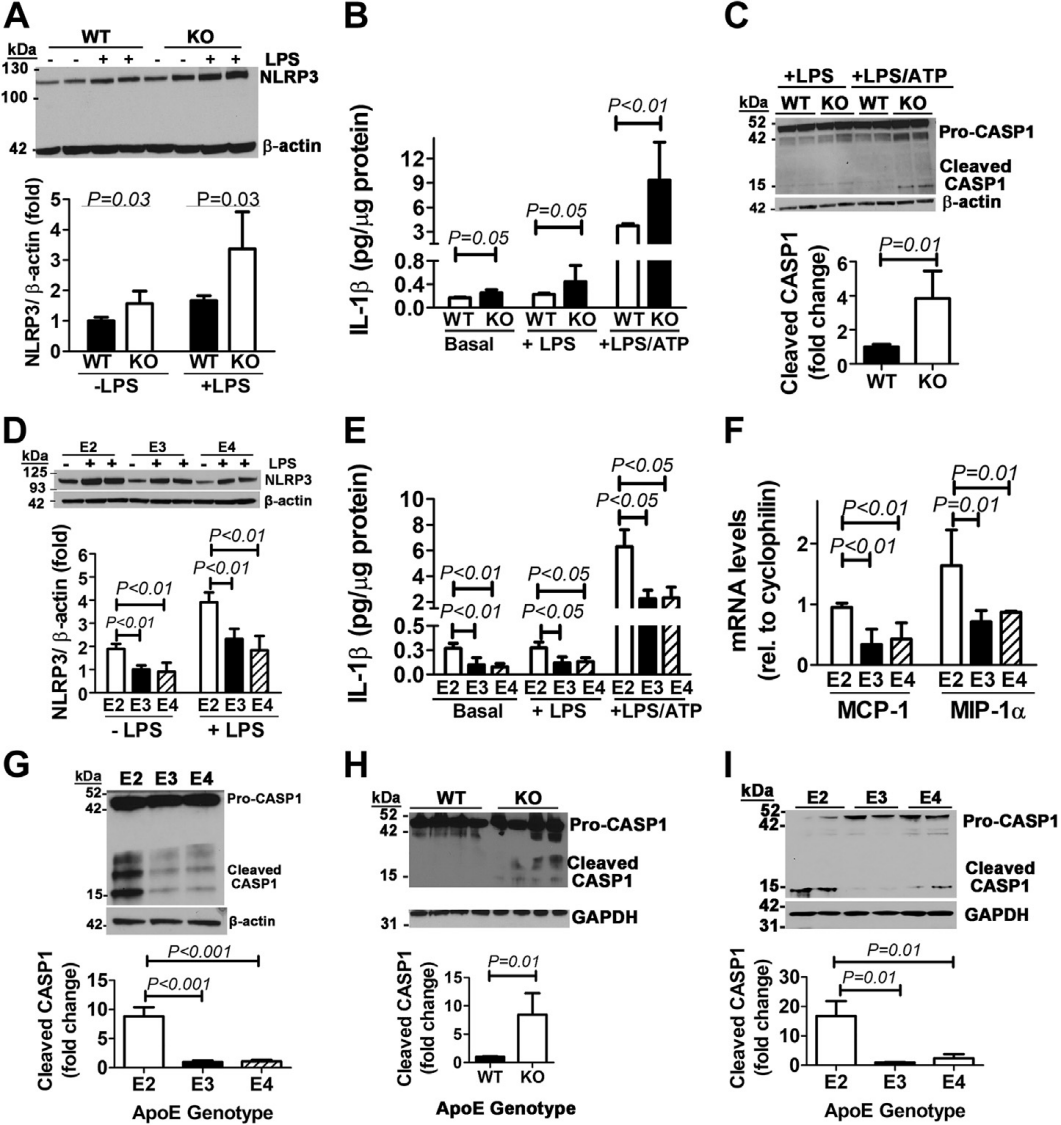
**ApoE deficiency or apoE2 expression in macrophages enhances inflammasome priming and activation.***A*, macrophages isolated from WT and *ApoE*^*−/−*^ (KO) mice (N = 4) were incubated in the absence (−) or presence of 100 ng/ml LPS. Extracts were prepared for Western blot analysis of NLRP3 levels using β-actin as the control. *B*, macrophages from WT and KO mice (N = 8) were incubated under basal conditions or in the presence of LPS with or without ATP stimulation. IL1-β secreted into the media was measured by ELISA. *C*, lysates prepared from WT and KO macrophages incubated with LPS with or without ATP were used for Western blot analysis with antibodies against caspase-1 (CASP1) to identify pro-caspase1 and its cleavage products as indicated. Similar experiments were performed with human *APOE2*, *APOE3*, and *APOE4* gene replacement mice, and results obtained include the following: (*D*) NLRP3 levels (N = 3); (*E*) IL-1β secretion under basal conditions (N = 10) or in the presence of LPS with (N = 6) or without (N = 7) ATP; (*F*) MCP-1 and MIP-1α mRNA levels in LPS-stimulated macrophages; and (*G*) caspase-1 cleavage analysis in LPS/ATP-stimulated macrophages. Extracts were also prepared from splenocytes isolated from 4 WT and four KO mice (*H*) or human *APOE2*, *APOE3*, and *APOE4* gene replacement mice (*I*) and subjected to Western blot analysis with antibodies against caspase-1 to identify pro-caspase1 and its cleavage product as indicated. All data were evaluated by one-way ANOVA with Student–Newman–Keuls post hoc test for significant differences between groups as indicated. apoE, apolipoprotein E; IL, interleukin; LPS, lipopolysaccharide; NLRP3, NOD-, LRR-, and pyrin domain–containing protein 3.

The enhancement of LPS-induced IL-1β and IL-18 secretion in the whole-blood assay is an indication of inflammasome activation. To test this possibility, the first experiment compared peritoneal macrophages isolated from WT and *ApoE*^*−/−*^ mice for inflammasome activation after incubation with or without LPS challenge. Indeed, NOD-, LRR-, and pyrin domain–containing protein 3 (NLRP3) levels and IL-1β secretion were found to be higher in *ApoE*^*−/−*^ macrophages under basal conditions as well as when challenged with LPS and ATP to induce inflammasome priming and activation (Fig. 2, *A* and *B*). Increased caspase-1 cleavage was also observed in LPS/ATP-activated *ApoE*^*−/−*^ macrophages compared with WT cells (Fig. 2*C*).

Similar to observations with apoE deficiency, levels of NLRP3 were also found to be higher in apoE2-expressing macrophages than apoE3- and apoE4-expressing macrophages under basal conditions without LPS stimulation (Fig. 2*D*). When the cells were incubated with LPS to prime macrophages, higher NLRP3 levels were observed in all three groups than unstimulated cells (Fig. 2*D*). Interestingly, the levels of NLRP3 expression in LPS-primed apoE3- and apoE4-expressing macrophages were similar to NLRP3 expression levels observed in apoE2-expressing macrophages under basal conditions (Fig. 2*D*), thus indicating that the macrophages from *APOE2* gene replacement mice were already primed for activation. Consistent with this interpretation, more IL-1β was found to be secreted by apoE2-expressing macrophages than apoE3- and apoE4-expressing macrophages under both basal and LPS-primed conditions (Fig. 2*E*), and IL-1β target genes such as monocyte chemoattractant protein-1 and macrophage inflammatory protein 1-α (48) were also increased in apoE2-expressing macrophages in response to LPS challenge (Fig. 2*F*).

The addition of ATP to the incubation medium of LPS-primed macrophages robustly enhanced IL-1β secretion in all three groups, with the greatest increase observed with apoE2-expressing macrophages (Fig. 2*E*). The addition of LPS and ATP also enhanced caspase-1 cleavage in *APOE2* macrophages compared with that observed in *APOE3* or *APOE4* macrophages (Fig. 2*G*).

The physiological significance of these *in vitro* observations was documented by higher levels of cleaved caspase-1 product in splenocytes of *ApoE*^*−/−*^ mice than those of WT mice (Fig. 2*H*) and in splenocytes of *APOE2* gene replacement mice than those of *APOE3* and *APOE4* gene replacement mice (Fig. 2*I*). Thus, the enhanced inflammatory response observed in *ApoE*^*−/−*^ and *APOE2* gene replacement mice is due to increased macrophage NLRP3 inflammasome priming and activation.

### Enhanced myelopoiesis in mice with apoE2-expressing myeloid cells

**Figure 3:**
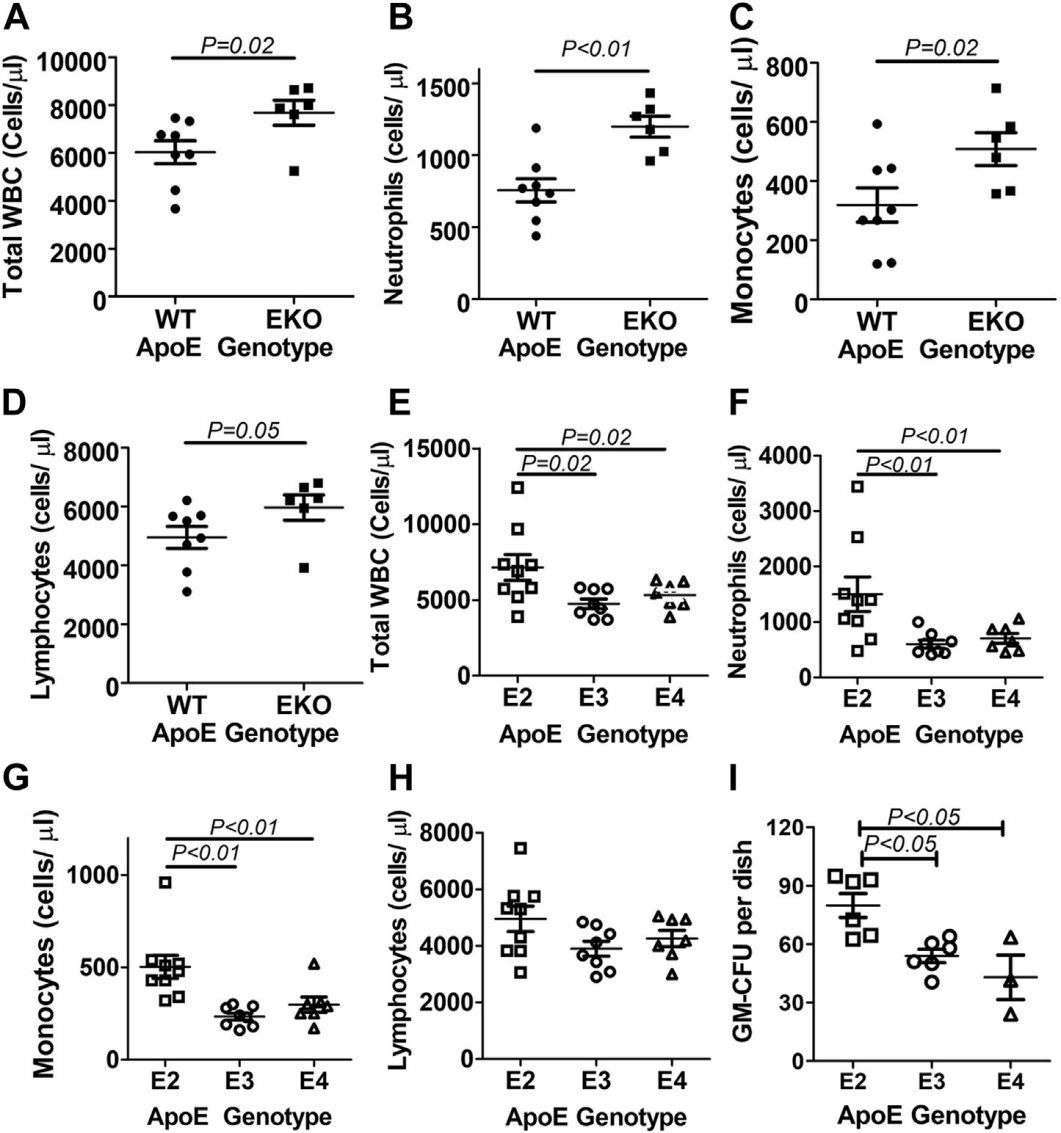
**Enhanced myelopoiesis in mice with apoE2-expressing myeloid cells.** Blood was obtained from WT and *ApoE*^*−/−*^ (EKO), *APOE2*, *APOE3*, and *APOE4* gene replacement mice (N = 8–10 in each group) for analysis by automatic white blood cell counter to determine the number of (*A*) total white blood cells in WT and KO mice; (*B*) neutrophils in WT and KO mice; (*C*) monocytes in WT and KO mice; (*D*) lymphocytes in WT and KO mice; (*E*) total white blood cells in *APOE2*, *APOE3*, and *APOE4* gene replacement mice; (*F*) neutrophils in the gene replacement mice; (*G*) monocytes in the gene replacement mice; and (*H*) lymphocytes in the gene replacement mice. *I*, bone marrow cells isolated from the gene replacement mice (N = 6 for apoE2 and apoE3 and N = 4 for apoE4 mice) were cultured for 7 days in the presence of cytokines to promote granulocyte/monocyte colony formation to determine GM-CFU. Statistical significance was evaluated by comparing WT and EKO mice with Student's *t* test. Comparisons between APOE gene replacement mice were evaluated by one-way ANOVA with Student–Newman–Keuls post hoc test. apoE, apolipoprotein E;

Increased NLRP3 inflammasome-dependent IL-1β production has been demonstrated to promote monocytosis and neutrophilia, associated with proliferation and expansion of bone marrow myeloid progenitors (49). Consistent with results reported in previous studies showing increased myelopoiesis and monocytosis in *ApoE*^*−/−*^ mice compared with WT control mice (38), analysis of blood leukocyte composition with an automatic cell counter showed the increase in the total leukocyte count as well as number of neutrophils, monocytes, and lymphocytes in *ApoE*^*−/−*^ mice compared with WT mice (Fig. 3, *A*–*D*). When the blood samples from the *APOE* gene replacement mice were analyzed, a significantly higher number of total leukocytes, particularly the myeloid-lineage neutrophils and monocytes but not the lymphoid-lineage lymphocytes, was found in *APOE2* gene replacement mice than *APOE3* and *APOE4* gene replacement mice (Fig. 3, *E*–*H*). In contrast, no significant differences in the number of blood cells were observed between *APOE3* and *APOE4* gene replacement mice (Fig. 3, *E*–*H*). Interestingly, flow cytometry analysis of lineage-negative bone marrow cells in the gene replacement mice revealed no differences in the number of myeloid precursors, such as long-term hematopoietic stem cell (HSC), multipotent progenitor-2, -3, and -4, common myeloid progenitor cell, or granulocyte-macrophage progenitor cell population among the three groups (data not shown). However, when bone marrow cells were isolated from the gene replacement mice and then incubated with cytokines that promote granulocyte/monocyte colony formation, a significantly higher number of granulocyte/monocyte colony-forming units (CFUs) was observed in apoE2-expressing bone marrow cells than apoE3- and apoE4-expressing cells after 7 days in culture (Fig. 3*I*). Taken together, these data suggest that apoE2 promotes monocytosis *via* multiple mechanisms including paracrine effects of NLRP3- and IL-1β-dependent mobilization of progenitor cells from the bone marrow to the peripheral blood (50) and IL-1β-mediated chemokine increase (48), as well as autocrine effect of increasing stem cell expansion.

### Cholesterol enrichment in apoE2-expressing myeloid cells

**Figure 4:**
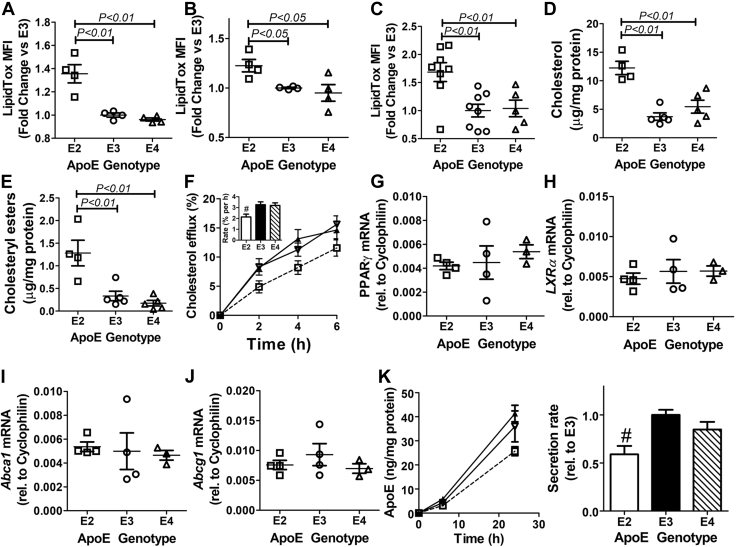
**Cholesterol enrichment in apoE2-expressing myeloid cells due to impairment of cholesterol efflux and apoE secretion.** The fluorescent dye LipidTOX was used to assess neutral lipid accumulation in (*A*) peripheral blood monocytes (N = 4), (*B*) peritoneal macrophages (N = 4), and (*C*) lineage-negative bone marrow cells (N = 8) isolated from *APOE2*, *APOE3*, and *APOE4* gene replacement mice. *D*, free cholesterol and (*E*) cholesteryl ester contents were measured in peritoneal macrophages isolated the gene replacement mice (N = 5) by biochemical methods. *F*, the peritoneal macrophages from *APOE2* (□, open bar), *APOE3* (▲, filled bar), and *APOE4* (Δ, hatched bar) were preloaded with [^3^H]cholesterol followed by incubation with HDL to assess cholesterol efflux. The data represent the mean ± SD using four mice per group. The *inset* shows the rate of cholesterol efflux calculated by linear regression. # indicates *p* < 0.05 difference from the other groups. mRNA was also isolated from macrophages of the gene replacement mice (N = 4) for amplification of PPARγ (*G*), LXRα (*H*), ABCA1 (*I*), and ABCG1 (*J*) mRNA. Expression levels were normalized to the cyclophilin mRNA levels in each sample. *K*, media were collected from cultured peritoneal macrophages from *APOE2* (□), *APOE3* (▲), and *APOE4* (Δ) mice (N = 6) for apoE quantification by ELISA. The graph on the *right* shows the rate of apoE secretion relative to the rate observed with apoE3-expressing cells. # indicates *p* < 0.05 difference from the other groups. All data were evaluated by one-way ANOVA with Student–Newman–Keuls post hoc test for significant differences as indicated. ABCA1, ATP-binding cassette-A1; ABCG1, ATP-binding cassette-G1; apoE, apolipoprotein E; HDL, high-density lipoprotein; LXRα, liver X receptor-α; PPARγ, peroxisome proliferator–activated receptor-γ.

Previous studies have shown that apoE-deficient mice have increased hematopoietic stem proliferation due to cholesterol accumulation as a consequence of impaired efflux from HSCs and myeloid cells (38). The assessment of intracellular neutral lipid levels by staining with the fluorescent dye LipidTOX revealed significantly more neutral lipids in peripheral blood monocytes, peritoneal macrophages, and lineage-negative bone marrow cells from *APOE2* gene replacement mice than those observed in *APOE3* or *APOE4* gene replacement mice (Fig. 4, *A*–*C*). Biochemical analysis revealed that increased levels of both free (unesterified) cholesterol and cholesteryl esters were responsible for the neutral lipid accumulation in apoE2-expressing macrophages (Fig. 4, *D* and *E*).

To determine whether neutral lipid accumulation in apoE2-expressing macrophages was due to impairment of intracellular cholesterol efflux, cholesterol-loaded macrophages from the gene replacement mice were incubated with high-density lipoproteins (HDLs) to initiate cholesterol efflux. Similar to results reported for apoE-deficient macrophages (38), macrophages from *APOE2* gene replacement mice also displayed an ∼1.5-fold reduction in the rate of cholesterol efflux compared with macrophages from *APOE3* and *APOE4* macrophages (Fig. 4*F*). The impairment of cholesterol efflux was not due to defective expression of genes in the macrophage cholesterol efflux pathway (51), as similar levels of peroxisome proliferator–activated receptor-γ, liver X receptor-α, ATP-binding cassette-A1, and ATP-binding cassette-G1 mRNA were found in *APOE2*, *APOE3*, and *APOE4* macrophages (Fig. 4, *G*–*J*). However, consistent with results observed with lentiviral vector–mediated apoE overexpression in macrophages (52), macrophages from gene replacement mice also displayed impairment of apoE2 secretion compared with that of apoE3 and apoE4 (Fig. 4*K*). In view of previous reports showing that apoE facilitates macrophage cholesterol efflux in an autocrine manner (37), and that apoE is required for cholesterol addition to small HDL to form cholesterol-enriched HDL (53), the impaired secretion of apoE2 is the likely cause for reduced cholesterol efflux and the resulting cholesterol accumulation in apoE2-expressing macrophages.

### Increased lipid rafts in apoE2- and apoE4-expressing macrophages

**Figure 5:**
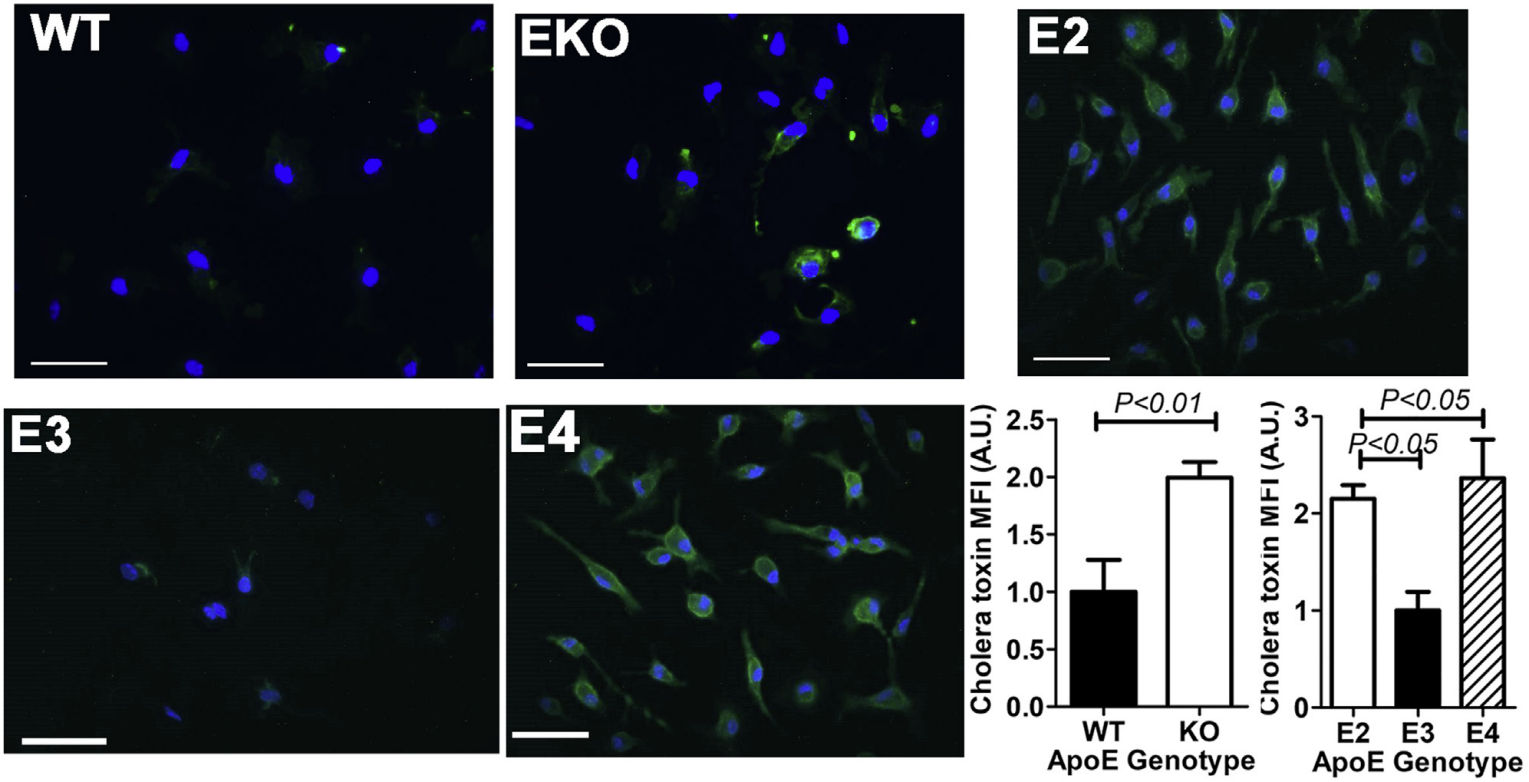
**Increased lipid rafts in apoE-deficient and apoE2- and apoE4-expressing macrophages.** Peritoneal macrophages isolated from WT, *ApoE*^*−/−*^ (EKO), *APOE2*, *APOE3*, and *APOE4* gene replacement mice were stained for GM1 with cholera toxin to identify lipid rafts. Representative images (scale bar = 50 μm) of data from four experiments are shown. Statistical significance was evaluated by comparing WT and EKO mice with Student's *t* test. Differences between *APOE* gene replacement mice were evaluated by one-way ANOVA with the Student–Newman–Keuls post hoc test. apoE, apolipoprotein E.

Increased cholesterol accumulation has been linked to increased plasma membrane lipid rafts. Therefore, we used cholera toxin staining of the glycosphingolipid GM1 to identify lipid raft domains in peritoneal macrophages isolated from WT, *ApoE*^*−/−*^, and the human *APOE* gene replacement mice. As expected, macrophages from *ApoE*^*−/−*^ mice exhibited higher lipid raft staining than WT mouse macrophages. ApoE2-expressing macrophages also displayed higher lipid raft staining than apoE3-expressing macrophages (Fig. 5). Surprisingly, apoE4-expressing macrophages also showed increased lipid raft staining despite similar intracellular cholesterol levels as apoE3-expressing macrophages (Fig. 5).

### ApoE4 but not apoE2 or apoE3 expression increases oxidative stress in macrophages

**Figure 6:**
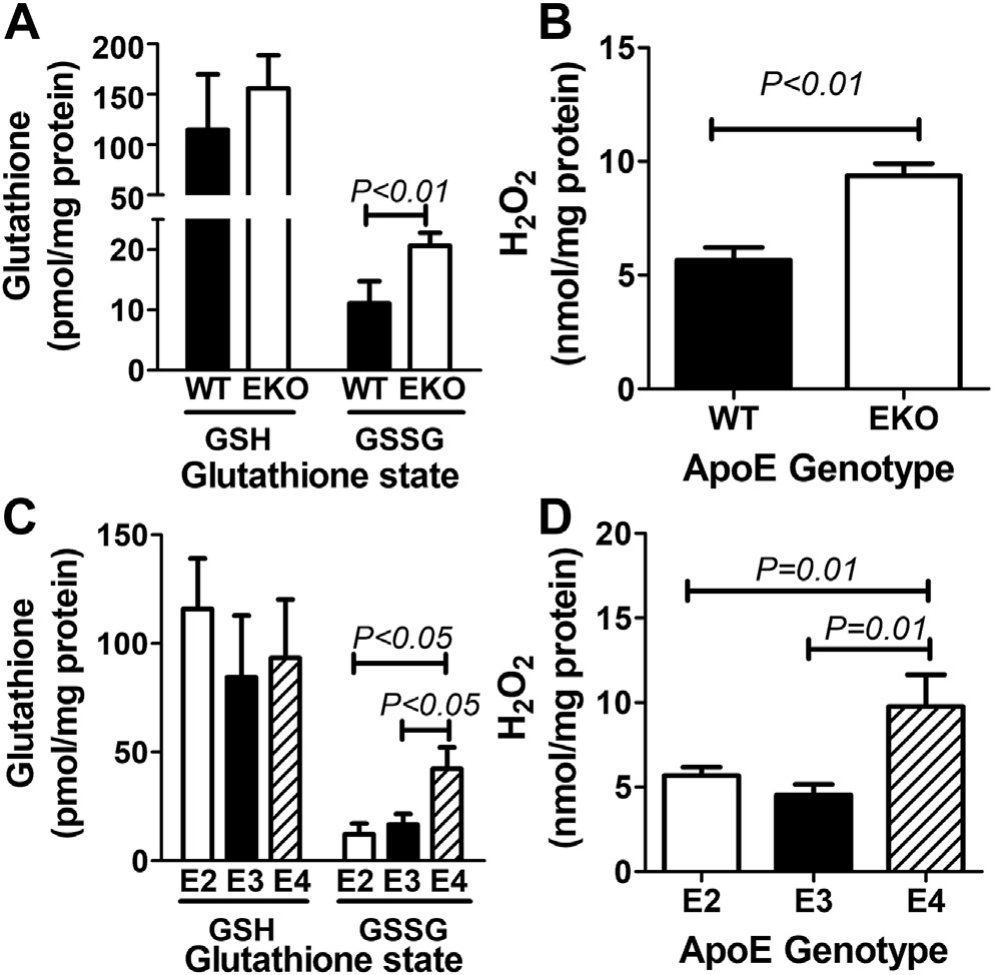
**Elevated oxidation status in peritoneal macrophages from *ApoE***^***−/−***^**and *APOE4* mice.** Peritoneal macrophages were isolated from WT, *ApoE*^*−/−*^ (EKO), *APOE2*, *APOE3*, and *APOE4* gene replacement mice (N = 7) to assess redox status by measuring reduced (GSH) and oxidized (GSSG) GSH levels and hydrogen peroxide levels. *A*, reduced GSH and oxidized GSH (GSSG) levels in WT and EKO macrophages; (*B*) hydrogen peroxide levels in WT and EKO macrophages; (*C*) GSH and GSSG levels in *APOE* gene replacement mice; and (*D*) hydrogen peroxide levels in *APOE* gene replacement mice. Statistical significance was evaluated by comparing WT and EKO mice with Student *t* test. Differences between *APOE* gene replacement mice were evaluated by one-way ANOVA with Student–Newman–Keuls post hoc test. apoE, apolipoprotein E.

Whereas the increased lipid raft levels in apoE2-expressing macrophages were likely due to elevated intracellular cholesterol levels as a consequence of cholesterol efflux impairment, the increased lipid raft levels in apoE4-expressing macrophages cannot be attributed to intracellular cholesterol content in the macrophages. One cholesterol-independent mechanism for lipid raft increase with enhanced toll-like receptor signaling is the reduction of membrane fluidity due to increased oxidative stress (54, 55). The measurement of GSH and GSSG levels as well as the level of intracellular hydrogen peroxide revealed increased oxidative stress in *ApoE*^*−/−*^ macrophages compared with WT macrophages (Fig. 6, *A* and *B*). Interestingly, increased levels of GSSG, the oxidized form of GSH, and H_2_O_2_ were also observed in apoE4-expressing macrophages but not apoE2- or apoE3-expressing macrophages (Fig. 6, *C* and *D*). These observations indicate that endogenously expressed apoE4 directly increased cellular oxidative stress, similar to the pro-oxidative properties of exogenously supplied apoE4 observed in numerous other studies (56). Importantly, the present study also showed that endogenously expressed apoE2 did not increase oxidative stress in macrophages, thereby highlighting the different mechanisms by which apoE2 and apoE4 exacerbate macrophage inflammatory response.

### ApoE isoforms differentially influence macrophage response to oxidized LDL

**Figure 7:**
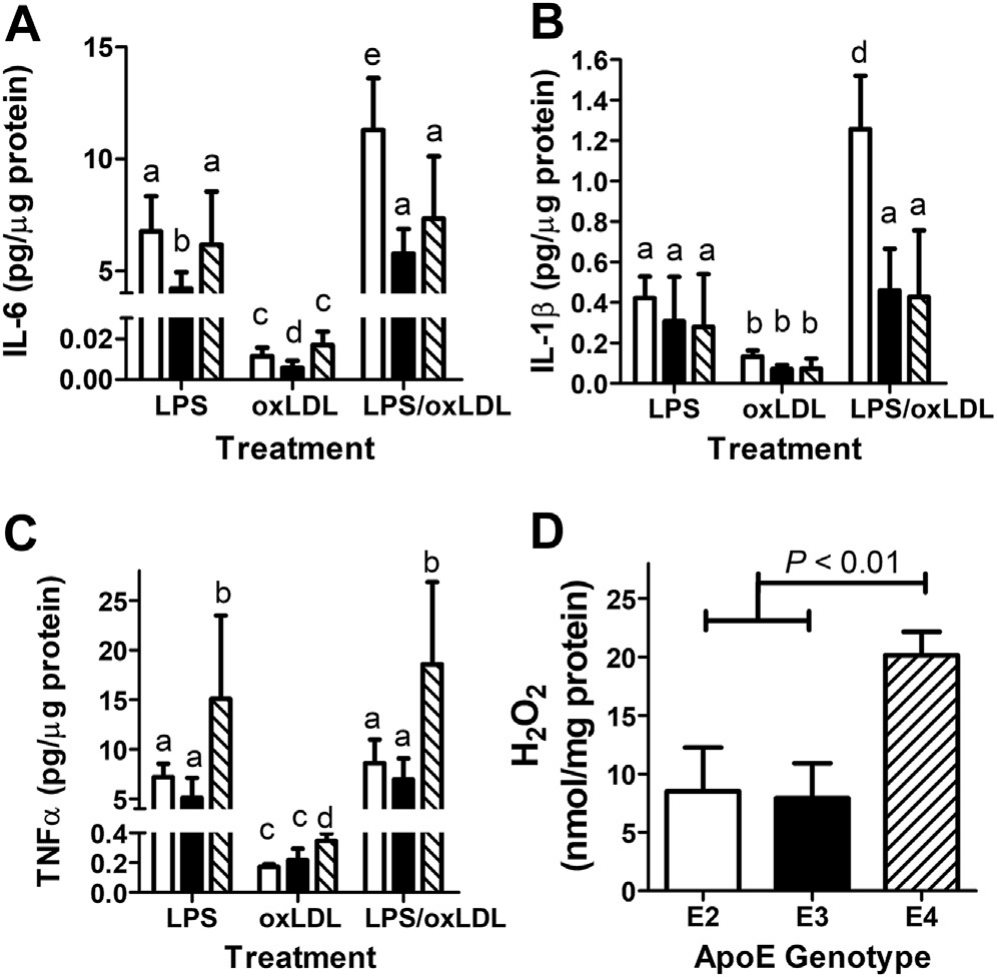
**Influence of *APOE* genotype on LPS and oxLDL stimulated cytokine secretion and oxidative stress in macrophages.** Macrophages isolated from *APOE2* (*open bars*), *APOE3* (*filled bars*), and *APOE4* (*hatched bars*) were stimulated by 4-h incubation with 100 ng/ml LPS (N = 4) or 50 μg/ml oxLDL (N = 6), or both (N = 6). Conditioned media were collected for ELISA assay of IL-6 (*A*), IL-1β (*B*), and TNFα (*C*). Lysates were collected from cells incubated with oxLDL (N = 4) for hydrogen peroxide measurements (*D*). All data were evaluated by one-way ANOVA with Student–Newman–Keuls post hoc test for significant differences between groups. *Bars* with *different letters* in panels *A*–*C* are significantly different at *p* < 0.05. apoE, apolipoprotein E; IL, interleukin; LDL, low-density lipoprotein; LPS, lipopolysaccharide; oxLDL, oxidized LDL; TNFα, tumor necrosis factor α.

Additional experiments were performed to determine whether macrophage response to oxidized LDL (oxLDL) is also apoE isoform dependent. In these experiments, macrophages isolated from *APOE2*, *APOE3*, and *APOE4* gene replacement mice were incubated with 50 μg/ml oxLDL for 24 h, and inflammatory cytokines secreted into the medium were compared with those observed with 4-h stimulation with 100 ng/ml LPS by the ELISA assay. Consistent with previous reports (57), macrophages incubated with oxLDL secrete minimal amounts of IL-6, TNFα, and IL-1β compared with LPS stimulation (Fig. 7, *A*–*C*). However, oxLDL addition to LPS-primed macrophages resulted in significantly higher IL-6 and IL-1β secretion by *APOE2* macrophages than LPS stimulation alone (Fig. 7, *A* and *B*). Interestingly, oxLDL did not exacerbate LPS-stimulated TNFα secretion regardless of the apoE isoforms expressed in the macrophages (Fig. 7*C*). Nevertheless, higher TNFα levels were found in the media of *APOE4* macrophages than that of *APOE2* and *APOE3* macrophages after stimulation with LPS with or without oxLDL (Fig. 7*B*). The higher levels of TNFα secretion by *APOE4* macrophages in response to LPS are likely due to increased toll-like receptor-4 signaling in lipid rafts (58). Indeed, we found that oxLDL incubation also resulted in higher hydrogen peroxide levels in *APOE4* macrophages than *APOE2* and *APOE3* macrophages (Fig. 7*D*).

### Altered lymphocyte composition in APOE2 and APOE4 gene replacement mice

**Figure 8:**
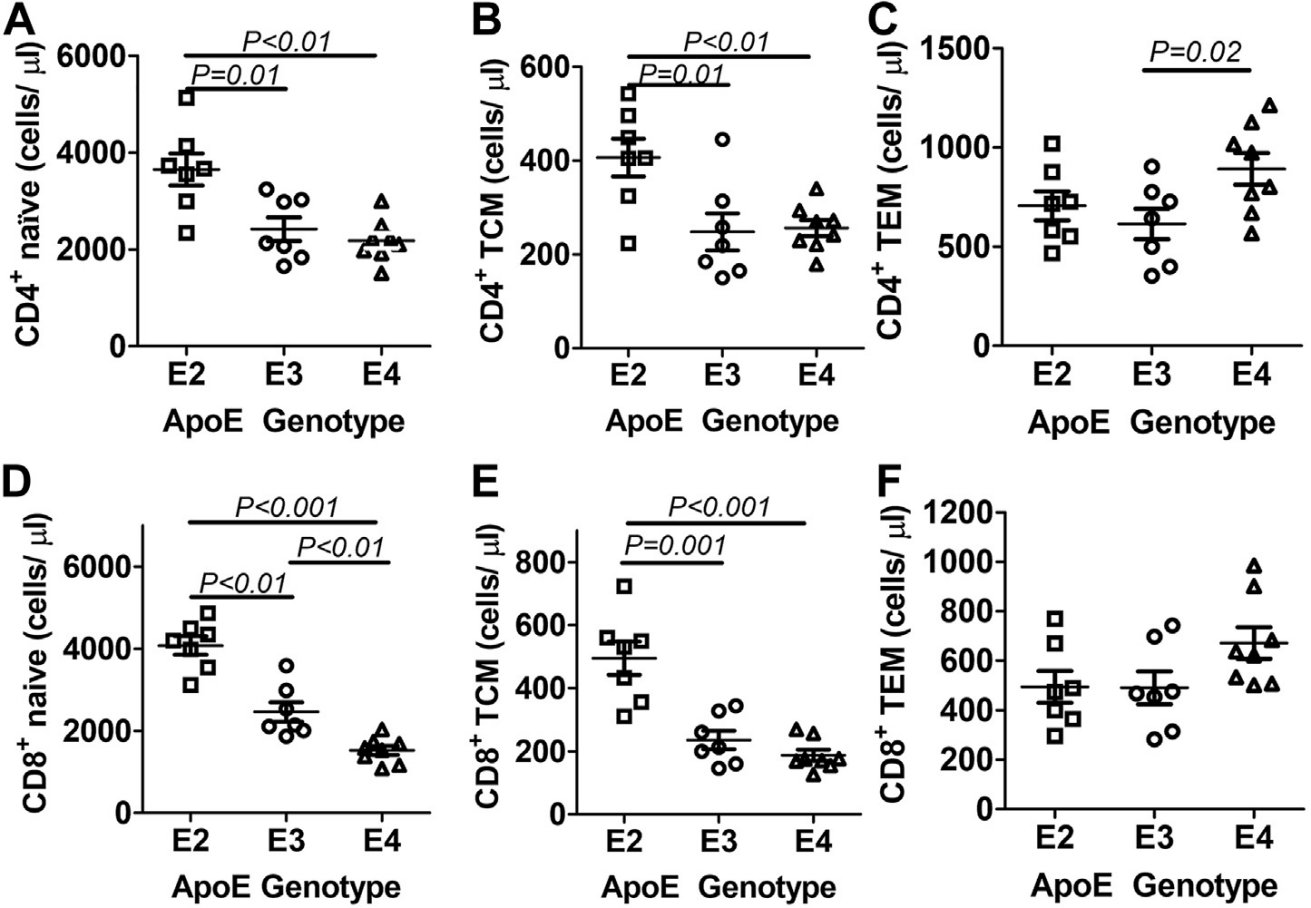
**Lymphocyte composition in the blood of *APOE* gene replacement mice.** Flow cytometry was used to analyze the number of (*A*) naïve CD4^+^ lymphocytes; (*B*) CD4^+^ T central memory cells; (*C*) CD4^+^ T effector memory cells; (*D*) naïve CD8^+^ T lymphocytes; (*E*) CD8^+^ T central memory cells; and (*F*) CD4^+^ T effector memory cells in the blood of *APOE2* (N = 7), *APOE3* (N = 7), and *APOE4* (N = 8) gene replacement mice. One-way ANOVA with Student–Newman–Keuls post hoc test was used to evaluate significant differences as indicated. apoE, apolipoprotein E.

ApoE is not expressed in lymphocytes (47, 59), but apoE expression in myeloid cells can influence adaptive immunity *via* modulation of antigen presentation. In particular, apoE deficiency or apoE4 expression in myeloid cells, particularly in dendritic cells, has been shown to enhance major histocompatibility complex II–dependent antigen presentation and CD4^+^ T cell activation (59), whereas apoE2 impairs lipid antigen presentation *via* the CD1d-mediated pathway (60, 61, 62). Therefore, we performed additional studies to assess the influence of apoE polymorphism on the adaptive immune system. Using flow cytometry to evaluate lymphocyte composition in the blood, we observed higher levels of naïve and central memory CD4^+^ T helper cells in the blood of *APOE2* gene replacement mice than those observed in the blood of *APOE3* and *APOE4* gene replacement mice (Fig. 8, *A* and *B*), but higher levels of CD4^+^ effector memory T cells were found in the circulation of *APOE4* mice (Fig. 8*C*). Higher levels of naïve and central memory CD8^+^ cytotoxic T cells were also found in *APOE2* gene replacement mice than *APOE3* and *APOE4* mice (Fig. 8, *D* and *E*), and less-naïve CD8^+^ T cells were observed in *APOE4* mice than in *APOE3* mice (Fig. 8*D*). No differences were observed in effector memory CD8^+^ cells regardless of the *APOE* genotype (Fig. 7*F*). Taken together, these results indicate that both *APOE2* and *APOE4* gene replacement mice are similar to human *APOE2* and *APOE4* carriers with elevated T cell activation compared with *APOE3* carriers (59), with increased number of CD4^+^ and CD8^+^ T cells in *APOE2* gene replacement mice and elevated CD4^+^ effector memory cells in *APOE4* gene replacement mice. Moreover, the lower number of naïve CD8^+^ T cells in *APOE4* mice may be due to enhanced CD8^+^ lymphocyte turnover in a pro-oxidation environment (63, 64).

### Bone marrow cells expressing human apoE3, but not apoE2 or apoE4, suppress atherosclerosis in ApoE^−/−^ mice

**Figure 9:**
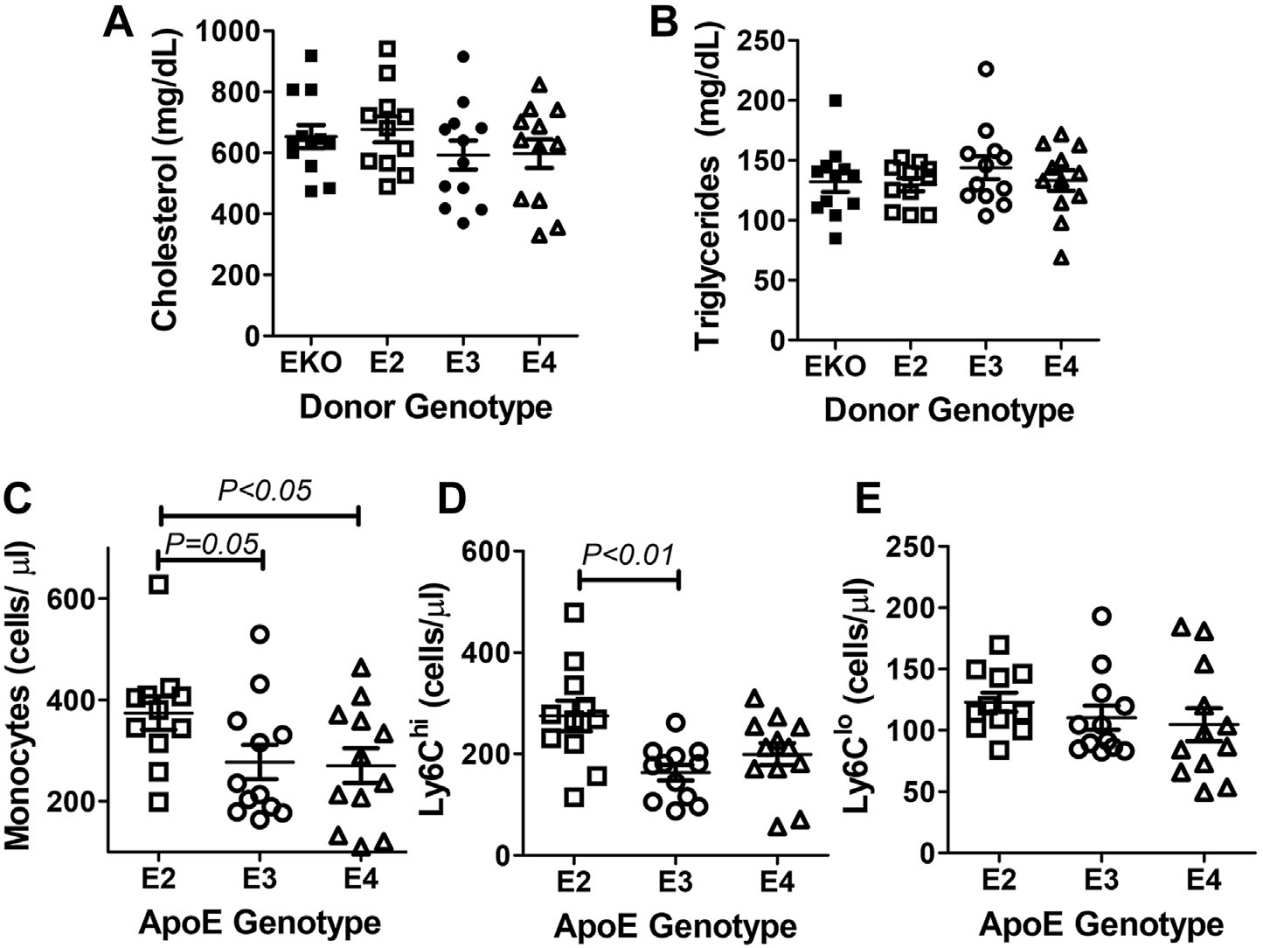
**Plasma lipid levels and monocyte subsets in *ApoE***^***−/−***^**mice after bone marrow transplants.** Lethally irradiated *ApoE*^*−/−*^ mice were transplanted with bone marrow cells from *ApoE*^*−/−*^ (EKO), *APOE2*, *APOE3*, and *APOE4* mice and then fed a Western diet for 8 weeks. *A*, plasma cholesterol and (*B*) triglyceride levels were determined from N = 12 mice in each group. Blood cells from the *APOE* gene replacement mice were obtained for flow cytometry analysis of (*C*) total monocytes, (*D*) Ly6C^hi^, and (*E*) Ly6C^lo^ monocytes. All data were evaluated by one-way ANOVA with the Student–Newman–Keuls post hoc test for statistical significance between groups as indicated. apoE, apolipoprotein E.

Inflammatory myeloid cells as well as CD4^+^ and CD8^+^ T cell activation are hallmarks of atherosclerosis (65, 66, 67, 68, 69). Therefore, the increased leukocyte inflammation observed in *APOE2* and *APOE4* gene replacement mice suggested that myeloid cell–derived apoE2 and apoE4 may be pro-atherogenic. To test this hypothesis, bone marrow cells isolated from human *APOE2*, *APOE3*, and *APOE4* gene replacement mice were transplanted into lethally irradiated *ApoE*^*−/−*^ mice to investigate how different apoE isoforms expressed endogenously in bone marrow cells may influence atherogenesis. Bone marrow cells isolated from *ApoE*^*−/−*^ mice were used as controls. The transplantation of bone marrows from the human *APOE* gene replacement mice did not change plasma lipid levels as similar plasma cholesterol and triglyceride levels were observed between Western diet–fed *ApoE*^*−/−*^, *APOE2*, *APOE3*, and *APOE4* bone marrow–recipient mice (Fig. 9, *A* and *B*). Interestingly, increased monocytosis was observed in *APOE2* bone marrow recipients compared with *APOE3* and *APOE4* recipients (Fig. 9*C*). In particular, the number of classical Ly6C^hi^ cells but not the nonclassical Ly6C^lo^ cells was found to be higher in the *APOE2* bone marrow–recipient mice (Fig. 9, *D* and *E*). Thus, the earlier observation of increased monocytosis in *APOE2* gene replacement mice was independent of the higher plasma lipid levels in these animals (70).

**Figure 10:**
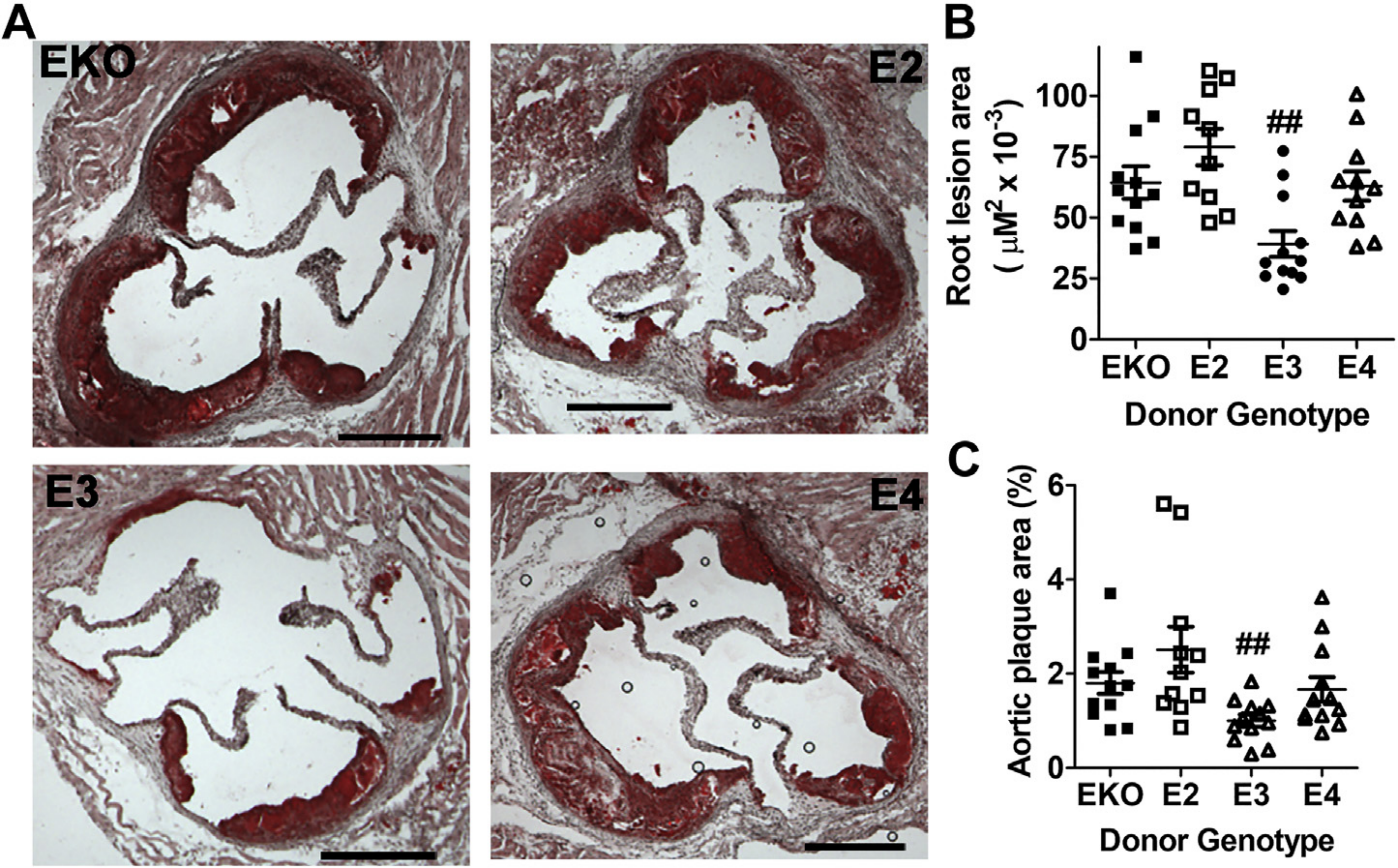
**Atherosclerosis lesion analysis in *ApoE***^***−/−***^**mice after bone marrow transplant.** Bone marrows obtained from *ApoE*^*−/−*^ (EKO), *APOE2*, *APOE3*, and *APOE4* mice were transplanted into irradiated *ApoE*^*−/−*^ mice and the animals were fed Western diet for 8 weeks. *A*, representative images (scale bar = 200 μm) of atherosclerotic lesions in aortic roots of EKO, *APOE2*, *APOE3*, and *APOE4* bone marrow recipients. *B*, morphometric data of lesion area in the aortic roots of 12 mice in each group were analyzed by one-way ANOVA with the Holm–Sidak post hoc analysis for comparisons between groups. ## indicates significant difference at *p* < 0.01 from the other groups. *C*, morphometric data of plaque area in the whole aorta from 12 mice in each group were analyzed for statistical significance similarly. ## indicates significant difference at *p* < 0.01 from the other groups. apoE, apolipoprotein E.

Evaluation of atherosclerosis in aortic roots and throughout the aorta of the bone marrow–recipient mice 8 weeks after feeding the Western diet showed that apoE3 bone marrow–recipient mice developed smaller atherosclerotic lesions in the aortic roots (Fig. 10, *A* and *B*) and the whole aorta (Fig. 10*C*) than recipients of apoE2, apoE4, or apoE-deficient bone marrow cells. These results indicate that human apoE3 expressed in bone marrow cells is similar to mouse apoE in suppressing atherogenesis, but myeloid cell–derived apoE2 and apoE4 are defective in protection against atherosclerosis in *ApoE*^*−/−*^ mice.

**Figure 11:**
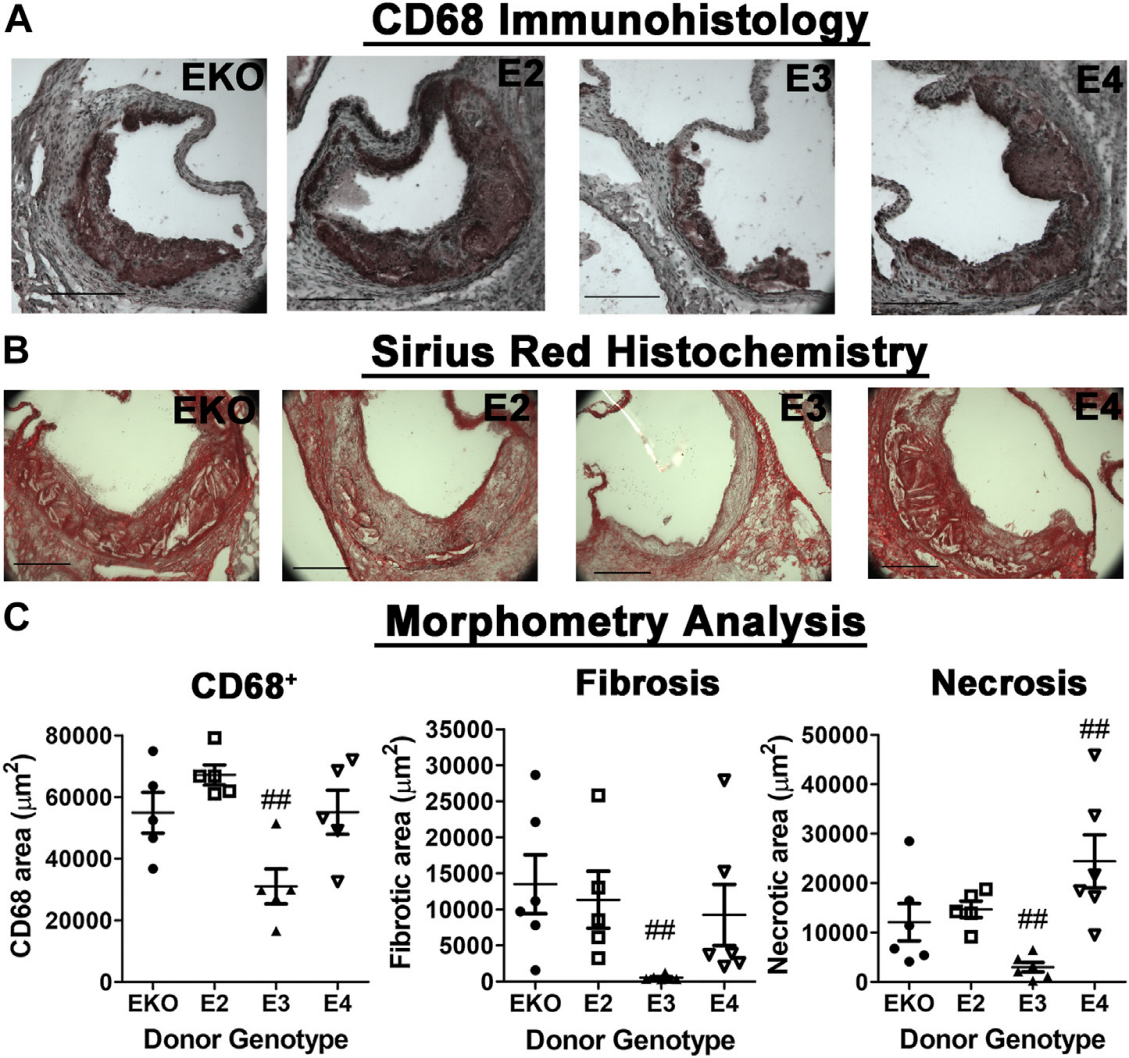
**Atherosclerosis composition in *ApoE***^***−/−***^**mice after bone marrow transplants.** Bone marrows obtained from *ApoE*^*−/−*^ (EKO), *APOE2*, *APOE3*, and *APOE4* mice were transplanted into irradiated *ApoE*^*−/−*^ mice, and the animals were fed Western diet for 8 weeks. Sections of the aortic roots were stained with (*A*) antibodies against CD68 to identify macrophages or (*B*) Sirius Red to identify fibrotic areas. The scale bar represents 200 μm. *C*, morphometric analysis of the data from N = 5 mice per group. The data were evaluated for statistical significance by one-way ANOVA with the Newman–Keuls multiple comparison test to identify differences between groups. ## indicates differences at *p* < 0.01 from the other groups. apoE, apolipoprotein E.

Immunohistological staining of the aortic root sections revealed that most of the lesion areas were occupied by CD68^+^ cells, with *APOE3* bone marrow–recipient mice displaying significantly less CD68^+^ cells compared with mice with *ApoE*^*−/−*^, *APOE2*, or *APOE4* bone marrow cells (Fig. 11, *A* and *C*). Sirius Red staining of the aortic root sections showed that the *APOE3* bone marrow–recipient mice also displayed less fibrotic areas (Fig. 11, *B* and *C*). Interestingly, examination of the Sirius Red–stained sections revealed dramatic differences in lesion necrosis after bone marrow transplant. Whereas the *APOE3* bone marrow–recipient mice displayed less lesion necrosis, the *APOE4* bone marrow–recipient mice displayed more necrotic lesions than mice with *ApoE*^*−/−*^ or *APOE2* bone marrow transplants (Fig. 11, *B* and *C*). Taken together, these results indicate that whereas apoE3-expressing bone marrow cells limit atherosclerosis progression, and atherosclerotic lesions in *ApoE*^*−/−*^ mice with *ApoE*^*−/−*^ and *APOE2* bone marrow cells were early-stage lesions after 8 weeks of Western diet feeding, the lesions in *APOE4* bone marrow–recipient mice have advanced to a more complex stage with cell necrosis.

**Figure 12:**
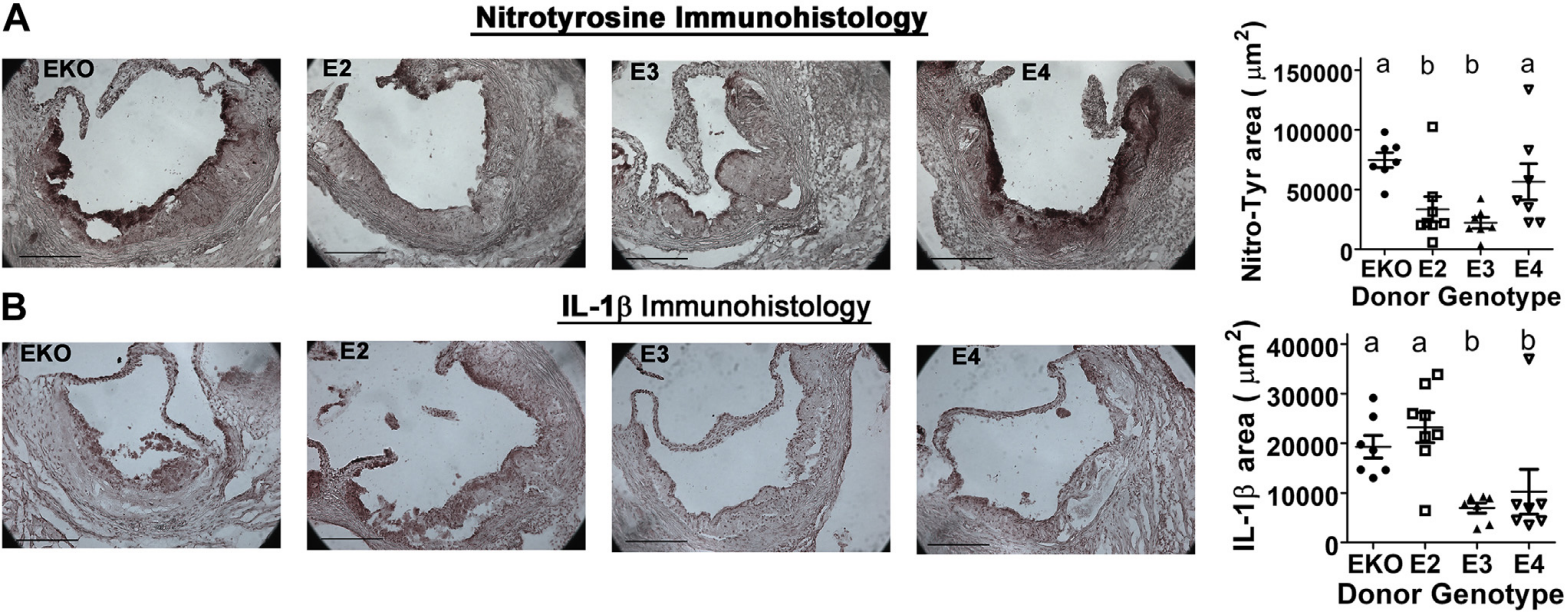
**Immunohistological analysis of nitrotyrosine and IL-1β epitopes in *ApoE***^***−/−***^**mice after bone marrow transplants.** Irradiated *ApoE*^*−/−*^ mice were transplanted with *ApoE*^*−/−*^ (N = 7), *APOE2* (N = 8), *APOE3* (N = 7), and *APOE4* (N = 7) bone marrow cells and fed Western diet for 8 weeks. Sections of the aortic roots were stained with antibodies against (*A*) nitrotyrosine or (*B*) IL-1β. The scale bar represents 200 μm. The data were evaluated for statistical significance by one-way ANOVA with the Newman–Keuls multiple comparison test to identify differences between groups. *Bars* with *different letters* indicate differences at *p* < 0.05. apoE, apolipoprotein E; IL, interleukin.

The increase in necrotic lesions in *APOE4* bone marrow–recipient mice despite the similar lesion size as in *ApoE*^*−/−*^ and *APOE2* bone marrow–recipient mice is likely due to increased oxidative stress in apoE4-expressing myeloid cells. Indeed, immunohistological staining of the nitrotyrosine epitope, a biomarker for peroxynitrite production and oxidative damage (71), revealed significantly higher levels of oxidative stress in the lesions of *APOE4* bone marrow–recipient mice (Fig. 12*A*). Interestingly, the level of IL-1β was found to be higher in the lesions of *ApoE*^*−/−*^ and *APOE2* bone marrow–recipient mice than *APOE3* and *APOE4* bone marrow–recipient mice (Fig. 12*B*). These observations corroborated well with the *in vitro* data showing that apoE4 promotes oxidative stress, whereas apoE2 increases inflammasome activation and IL-1β production.

## Discussion

This study used human *APOE2*, *APOE3*, and *APOE4* gene replacement mice, expressing different human apoE isoforms under the control of physiologically regulated mouse *ApoE* gene promoter, and showed distinct mechanisms by which each apoE isoform expressed in myeloid lineage cells influences inflammation and atherogenesis. Our results showed that whereas both apoE2 and apoE4 expressed in myeloid cells are pro-inflammatory, similar to cells with apoE deficiency, the mechanisms underlying the pro-inflammatory properties of apoE2 and apoE4 are quite different. Specifically, apoE2 promotes inflammation *via* inflammasome activation and apoE4 promotes inflammation through increased oxidative stress. Nevertheless, bone marrow cells expressing human apoE2 and apoE4 are similar to apoE-deficient cells in their ineffectiveness to suppress atherosclerosis in Western diet–fed *ApoE*^*−/−*^ mice, whereas bone marrow cells expressing human apoE3 were atheroprotective when transplanted into *ApoE*^−/−^ mice. It is noteworthy that all four groups of bone marrow recipients displayed similar hypercholesterolemia, thus documenting that the differences in atheroprotection were not due to differences in plasma lipoprotein clearance in *ApoE*^*−/−*^ mice.

The *ex vivo* whole-blood assay revealed that in comparison to *APOE3* gene replacement mice, the *APOE2* gene replacement mice were more sensitive to agonist-induced inflammatory response (Fig. 1). The *APOE2* gene replacement mice also displayed increased number of myeloid cells in circulation (Fig. 3), and their myeloid cells as well as HSCs were enriched with intracellular cholesterol because of cholesterol efflux impairment (Fig. 4). The cholesterol enrichment and impairment of cholesterol efflux resulted in enhanced myelopoiesis and monocytosis (38, 72, 73, 74), thereby increasing the myeloid cell number in *APOE2* gene replacement mice. Increased intracellular cholesterol content due to cholesterol efflux impairment has also been reported to increase inflammasome priming and activation (75, 76, 77). Indeed, increased inflammasome priming and activation was evident in peritoneal macrophages as well as splenocytes isolated from *APOE2* gene replacement mice (Fig. 2). The increased inflammasome priming and activation observed with apoE2-expressing peritoneal macrophages indicated that the observed increased *ex vivo* response of the blood cells from *APOE2* gene replacement to agonist-induced activation was due to not only increased myeloid cell number in the blood but also a direct effect of increased inflammatory response of apoE2-expressing myeloid cells. Taken together, the collective results indicate that apoE2 enhances inflammation through a combination of increased myelopoiesis and elevated inflammatory response of the myeloid cells. These pro-inflammatory properties preclude the ability of myeloid-derived apoE2 to suppress atherosclerosis.

The immunoregulatory properties of apoE2 have been reported previously, with opposing results that appeared to be cell type dependent. In microglia, apoE2 was shown to be similar to apoE3 in limiting inflammatory response to LPS (78). In contrast, increased pro-inflammatory cytokine production was observed in apoE2-expressing astrocytes (79). While these studies did not explore the underlying mechanism by which apoE2 differs in regulating inflammatory response in microglia and astrocytes, it is interesting to note that IL-1β expression was lower in LPS-stimulated microglia but was enhanced in LPS-stimulated astrocytes (78, 79). In the present study, we confirmed the proinflammatory properties of apoE2 in macrophages (44) and extended on these previous studies with results indicating that the mechanism is due to enhanced inflammasome priming and activation, similar to that observed in astrocytes. The cause of the elevated inflammasome priming observed in apoE2-expressing macrophages is likely due to higher intracellular cholesterol level as a consequence of impaired apoE secretion and cholesterol efflux from the cells. The impaired secretion of endogenously synthesized apoE2 is cell type dependent (52), thus suggesting that the cell-specific anti- or pro-inflammatory properties of human apoE2 are dependent on its secretion or accumulation of intracellular cholesterol in each cell type.

This study showed that apoE4-expressing myeloid cells also exhibited an exaggerated proinflammatory response with increased IL-6 and TNFα secretion after LPS stimulation. In contrast to apoE2-expressing cells, the apoE4-expressing macrophages did not show enhanced IL-1β and IL-18 response, thus the pro-inflammatory phenotype of apoE4-expressing macrophages is independent of NLRP3 inflammasome activation. The difference between apoE2 and apoE4 in macrophage inflammasome priming and activation is likely due to the normal apoE4 secretion that accompanied cholesterol efflux, thereby limiting intracellular cholesterol accumulation and priming of the inflammasome complex. The lower intracellular cholesterol content in apoE4-expressing hematopoietic cells than apoE2-expressing cells also resulted in limiting myelopoiesis to levels observed in apoE3-expressing cells. Hence, the pro-atherogenic and pro-inflammatory properties of myeloid-derived apoE4 are different from those of apoE2 and limited to inflammasome-independent inflammatory response without enhanced HSC proliferation and monocytosis.

The pro-inflammatory properties of apoE4 are well documented in the literature (56). In particular, the higher production of pro-inflammatory cytokines by apoE4-expressing macrophages is due to transactivation of the redox-sensitive transcription factor NF-kappa B (80). Consistent with these earlier findings, we showed higher levels of GSSG and hydrogen peroxide in macrophages isolated from *APOE4* gene replacement mice than those in apoE2- and apoE3-expressing macrophages. The increased oxidative stress is correlated with intracellular cholesterol-independent increase in lipid raft structures in the apoE4-expressing macrophages. In view of the critical role of lipid rafts in toll-like receptor-4–mediated inflammatory cell signaling (58), and the culmination of TLR signals in the activation of NF-kappa B (81), our results establish a link between the apoE4 expression and the NF-kappa B-directed inflammatory response in macrophages.

This study also showed differences in T cell composition among the three groups of human apoE gene replacement mice. In comparison to mice expressing the normal apoE3 isoform, CD4^+^ and CD8^+^ T cell levels were found to be higher in *APOE2* gene replacement mice but lower in the *APOE4* gene replacement mice. In contrast, the *APOE4* gene replacement mice contained significantly more CD4^+^ T effector cells than *APOE2* and *APOE3* mice. It is important to note that apoE is not expressed in lymphocytes (47, 59), thus suggesting that the differences in lymphocyte subsets may be due to the different apoE isoforms expressed in myeloid cells. A recent study reported that dendritic cells from human carriers of the ε4 allele displayed increased lipid raft clustering in dendritic cells and caused enhanced antigen presentation and T-cell activation (59). Thus, the elevated levels of T effector cells observed in *APOE4* gene replacement mice are likely due to enhanced antigen presentation by apoE4-expressing myeloid cells. A mechanistic difference between the earlier study and our results is that higher cholesterol levels were noted in the apoE4-expressing dendritic cells, and the investigators attributed the enhanced T cell activation to impaired cholesterol efflux to apoE4-containing HDL in the plasma. In contrast, our results showed that cholesterol levels in the myeloid and HSCs of *APOE4* mice were not elevated and were similar to that observed in *APOE3* gene replacement mice. Despite these differences, which remain to be resolved, increase in lipid raft structures in apoE4-expressing myeloid cells was also observed in our study, but we attributed the increased lipid rafts to apoE4-induced oxidative stress.

The results showing that bone marrow cells expressing the normal human apoE3 isoform are effective in suppressing atherogenesis in *ApoE*^*−/−*^ mice are consistent with previous studies demonstrating atheroprotection in *ApoE*^*−/−*^ mice with bone marrow cells expressing mouse apoE (39, 41). However, our results showed that reconstitution with apoE3-expressing bone marrow cells did not improve hypercholesterolemia in Western diet–fed *ApoE*^*−/−*^ mice, which are in striking contrast to previous studies showing that reconstitution with mouse apoE–expressing bone marrow cells reduced plasma cholesterol level (39, 41). These differences may be due to species-specific differences between mouse apoE and human apoE3. An independent study reporting that macrophage-specific human apoE3 transgenic expression reduced atherosclerosis in *ApoE*^*−/−*^ mice without hypercholesterolemia improvement is consistent with this interpretation (40). Nevertheless, regardless of potential differences between bone marrow–derived human and mouse apoE in regulating plasma cholesterol levels in *ApoE*^*−/−*^ mice, our results showed that bone marrow cell–derived human apoE3 is effective in suppressing atherogenesis without hypercholesterolemia improvement, thus documenting a lipoprotein transport–independent function of myeloid cell–derived apoE3 in atheroprotection.

In contrast to bone marrow cells expressing human apoE3, reconstitution of lethally irradiated *ApoE*^*−/−*^ mice with bone marrow from *APOE2* gene replacement mice was ineffective in atheroprotection. The failure of bone marrow cells from *APOE2* gene replacement mice to limit atherogenesis in *ApoE*^*−/−*^ mice is consistent with previous reports showing that bone marrow cells from transgenic mice with human *APOE2* overexpression were also ineffective in atheroprotection (41). However, our studies differ in that impaired cholesterol efflux was observed in our study with *APOE2* gene replacement mouse macrophages but human apoE2 overexpression was capable of promoting cholesterol efflux (41). While the differences in cholesterol efflux between the two studies are likely due to different levels of apoE2 expression in the macrophages, both studies showed that myeloid-derived apoE2 is ineffective in atheroprotection. Thus, in addition to its defect as an extracellular protein in binding LDL receptors and plasma lipoprotein clearance, the arginine-to-cysteine mutation at residue 158 in apoE2 also impairs the lipoprotein transport–independent atheroprotective functions of apoE.

In summary, our studies established apoE isoform–specific differences in the regulation of immune response and atherogenesis in a manner that is independent of plasma lipoprotein metabolism. While apoE2 and apoE4 are both pro-inflammatory in myeloid cells and apoE2- and apoE4-expressing bone marrow cells are pro-atherogenic, the underlying mechanisms by which these apoE isoforms enhance inflammation and atherosclerosis are quite different. Specifically, apoE2-enhanced inflammation is due to impaired cholesterol efflux, leading to elevated myelopoiesis and inflammasome priming and activation, whereas the apoE4-enhanced inflammation is due to elevated oxidative stress and T cell activation. The distinct mechanisms by which each apoE isoform influences myeloid cell functions are reminiscent of isoform-specific differences in signaling pathways in neurons that affect Alzheimer’s disease (82). However, whereas apoE2 expression in neurons appears to be protective against Alzheimer’s disease, this study showed that apoE2 expressed in myeloid cells is pro-inflammatory and pro-atherogenic.

## Experimental procedures

### Antibodies and primers

All antibodies and primers used in this study were obtained from commercial sources as listed in Tables S1 and S2.

### Animals and diets

Gene replacement mice, in which the endogenous murine *ApoE* gene has been replaced with the human *APOE2*, *APOE3*, or *APOE4* gene at the same locus, were originally generated in the Maeda laboratory (83, 84). These animals have been imported into our institution, backcrossed to C57BL/6J background, and maintained in our facility for many years (70, 85). The *ApoE*^*−/−*^ mice, also in C57BL/6J background, were purchased from the Jackson Laboratory. Mice were housed in our institutional animal care facility with free access to normal chow diet (LM485; Harlan-Teklad) and water. All procedures and animal care techniques were performed under a protocol approved by the Institutional Animal Care and Use Committee at the University of Cincinnati.

### *Ex vivo* whole-blood cytokine secretion assay

Blood was collected from mice after a 16-h fast. The blood was mixed with an equal volume of the RPMI medium containing LPS to a final concentration of 100 ng/ml and incubated at 37 °C for 4 h. The blood was then collected for centrifugation to separate the plasma/medium supernatant from the blood cells. Cytokines secreted into the plasma/medium were quantified using the following ELISA kits according to manufacturer’s instructions: IL-6 (M6000B), IL-18 (7625), and IL-1β (MHSLB00) from R&D Systems and TNFα (BMS607-3) from Thermo Fisher Scientific.

### Blood chemistry and cell count

Mice were fasted for 16 h before collection of blood from the submandibular vein into EDTA-coated tubes and kept on ice. Samples were centrifuged at 2500*g* for 10 min to collect plasma for triglyceride and cholesterol measurements with the Infinity colorimetric assay kits (TR22421 and TR13421, Thermo Fisher Scientific). For leukocyte cell count, blood was briefly warmed to room temperature (RT) and mixed thoroughly before analysis using an automatic white blood cell counter (Hemavet 950, Drew Scientific).

### Granulocyte–monocyte CFU assay

Bone marrows from *APOE2*, *APOE3*, and *APOE4* gene replacement mice were harvested and subject to erythrocyte lysis before plating 2 × 10^4^ cells in 1 ml of methylcellulose-based medium (MethoCult GF M3534, STEMCELL Technologies) per well in a 6-well plate according to manufacturer’s instructions. The number of CFU per dish was counted after 7 days.

### Peritoneal macrophage isolation

Mice were injected intraperitoneally with 1 ml of 4% thioglycolate broth to elicit peritoneal macrophages. The mice were euthanized after 3 days, and the peritoneal cavities were lavaged with 10 ml of ice-cold PBS. Peritoneal cells were collected and then centrifuged at 500*g* for 5 min at 10 °C, washed once, and plated in a cold RPMI medium supplemented with 10% FBS. Nonadhering cells were washed off after 1 h, and the macrophages adhered to the plates were used for experiments.

### Macrophage secretion

Macrophages were seeded in 6-well plates at a density of 2 × 10^6^ cells per well. For cytokine secretion measurements, cells were pretreated with the fresh serum-free RPMI medium with or without 100 ng/ml LPS for 4 h, followed by treatment with 2 mM ATP for 30 min or 50 μg/ml oxLDL for 24 h. For apoE secretion measurements, macrophages were cultured in the serum-free RPMI containing 0.2% fatty acid–free bovine serum albumin (BSA), and the samples were collected after 6 and 24 h. ELISA kits were used to measure human apoE in the medium (3712-1HP, Mabtech). The concentrations of IL-1β (MLB00C, R&D Systems), TNFα (BMS607-3, Thermo Fisher Scientific), and IL-6 (M6000B, R&D Systems) were also determined by ELISA.

### Intracellular cholesterol measurement

Macrophages were homogenized in 0.5 ml solution containing 50 mM Tris HCl, pH 7.4, 150 mM NaCl, and 5 mM EDTA. The homogenates were mixed with an equal volume of petroleum ether, vortexed, and centrifuged at 1000*g* for 10 min for phase separation. Lipid extraction was performed two times, and the lipid phase was combined, dried, and resuspended in the assay buffer for measurement of free and total cholesterol using Amplex Red kit (A12216, Life Technologies). Cholesteryl ester level was determined by subtracting the amount of free cholesterol from the total cholesterol content in each sample.

### Macrophage cholesterol efflux

Mouse peritoneal macrophages were seeded in 48-well plates at a density of 3 × 10^5^ cells per dish. The macrophages were induced to accumulate cholesterol by incubation overnight in the RPMI medium containing 0.2% fatty acid–free BSA and 50 μg/ml acetylated LDL that was previously labeled with 0.5 μCi/ml [^3^H]cholesterol. The radiolabel-containing medium was removed by washing twice with PBS. The cells were allowed to equilibrate in the phenol red–free RPMI medium with 0.2% BSA for 2 h before incubation with or without 20 μg/ml HDL to stimulate cholesterol efflux. [^3^H]cholesterol secreted into the medium was sampled at 2-h intervals for 6 h. The remaining cell lipids were extracted with isopropanol. [^3^H]cholesterol in the conditioned media and cell extracts were measured by scintillation counting, and the percent cholesterol efflux to HDL was calculated as (counts per minute in medium/counts per minute in cell extracts) × 100. Radioactivity in the media of cells incubated without HDL was used as baseline controls.

### Lipid raft estimation

Cellular lipid raft levels were estimated based on staining of GM1 with cholera toxin stain. The cells seeded on glass coverslips and cultured overnight were washed with PBS and then incubated with 4 μg/ml FITC-conjugated cholera toxin (C1655, Sigma) in the ice-cold RPMI medium for 30 min in the dark. Cells were washed three times with PBS and fixed for 15 min in 4% paraformaldehyde. Cell signals were quenched with 50 mM NH_4_Cl and then counterstained with DAPI. The coverslips were mounted with Gelvatol containing DABCO and visualized by fluorescence microscopy. Cholera toxin staining was quantified using ImageJ software.

### Intracellular redox assessment

Peritoneal macrophages were lysed in ice-cold 0.5% NP-40 in PBS. Samples were deproteinated using a trichloroacetic acid precipitation kit (ab204708, Abcam). Intracellular GSH and hydrogen peroxide levels were measured using fluorescent assay kits (ab205811 and ab102500, respectively, Abcam).

### Flow cytometry analysis of bone marrow cells and blood leukocytes

Lineage-negative bone marrow cells were enriched using negative selection with antibody-coated Dynabeads (11429D, Life Technologies Cabinet) and washed in the flow cytometry staining buffer containing Ca^2+^/Mg^2+^-free Hanks’ balanced salt solution with 0.3% NaN_3_ and 1% BSA. The cells were stained with stem cell and progenitor markers to identify long-term HSCs (Sca-1^+^c-kit^+^CD150^+^CD48^−^CD34^−^) and multipotent progenitor cells (Sca-1^+^c-kit^+^CD150^+/−^CD48^+/−^, CD34^+^) or common myeloid progenitor (Sca-1^+^c-kit^+^CD34^int^CD16/32^int^) and granulocyte-macrophage progenitor (Sca-1^+^c-kit^+^,CD34^int^CD16/32^hi^) cells as previously described (86). For the analysis of blood leukocytes, blood was collected from 16-h fasted animals following brief red blood cell lysis, blocked with CD16 and CD32 antibodies and then stained with Ly6G, Ly6C, CD11b, and CD115 antibodies to identify neutrophils (Ly6G^+^CD11b^+^) and monocytes (Ly6G^−^Ly6C^+^CD11b^+^CD115^+^) in the myeloid lineage. To assess intracellular neutral lipid accumulation, bone marrow cells, peritoneal macrophages, and blood leukocytes were stained with HSC LipidTOX (Invitrogen, H34475) at 1:10,000 dilution in flow cytometry staining buffer.

For the determination of lymphocyte subsets, the cells were stained with CD3, CD4, CD8, CD62L, and CD44 antibodies for flow cytometry analysis. Specific antibodies used for all flow cytometry analyses are listed in Table S1. Flow cytometry was performed using Guava easyCyte 8HT System and analyzed using Guava InCyte (Millipore). Gating strategies used to identify lymphocyte subsets are shown in Fig. S1.

### Lipoprotein isolation and preparation

Fresh human plasma obtained from the Hoxworth Blood Center was used for ultracentrifugal flotation of LDLs and HDLs (87). Acetylated LDL was prepared by mixing 5 mg/ml LDLs with an equal volume of ice-cold saturated sodium acetate with addition of 2-μl acetic anhydride four times over a 1-h period with continuous mixing on ice. oxLDL was prepared as previously described (85). All lipoprotein solutions were dialyzed extensively in PBS before use in experiments.

### Bone marrow transplantation and atherosclerosis studies

Ten-week-old male *ApoE*^*−/−*^ mice were lethally irradiated with two doses of 450 rad 4 h apart using a XenX closed-cabinet X-ray irradiator. The irradiated *ApoE*^*−/−*^ mice were reconstituted with 10 × 10^6^ bone marrow cells harvested from the tibia and femurs of 10- to 13-week-old male *ApoE*^*−/−*^, *APOE2*, *APOE3*, or *APOE4* gene replacement mice the following day. The bone marrow–recipient mice were maintained on a normal chow diet and administered 2 mg/ml neomycin antibiotic (Sigma) in the drinking water 1 week before and 4 weeks after irradiation to allow for recovery. The mice were then fed the Western type diet containing 41% fat and 0.2% cholesterol (D12331, Research Diets) for 8 weeks before tissue harvesting to evaluate atherosclerosis.

### Atherosclerotic lesion analysis

Anesthetized mice were perfused with 10% formalin in buffered saline for 5 min before dissection of the heart and the entire aorta to the iliac bifurcation. The tissues were stored in 10% buffered formalin solution for 1 week. The aortas were opened longitudinally and stained with Oil Red O for 30 min, whereas the top half of the heart was cryopreserved in 4% paraformaldehyde at 4 °C overnight before imbedding in OCT compound for frozen section preparation. Eight cryosections of 7-μm thickness through the aortic valve region were stained with Oil Red O for 15 min and counterstained with hematoxylin for 3 min. Composition of the atherosclerotic lesions was determined by staining serial sections for 1 h with Sirius Red to identify fibrotic areas and immunohistochemical analysis with antibodies against CD68, IL-1β, or nitrotyrosine. Immunohistochemical analysis was performed using VECTASTAIN ABC-HRP kits (Vector Laboratories) according to manufacturer's instructions. Images were obtained using an Olympus BX6 microscope and digitalized for quantitative analysis using ImageJ software as described (88).

### Western blot

Cells were homogenized in ice-cold RIPA buffer containing 50 μM Tris HCl, pH 7.4, 150 mM NaCl, 0.5% sodium deoxycholate, 1% igepal, 0.1% SDS, 1 mM EDTA, 1× phosphatase inhibitor cocktail 2 (Sigma-Aldrich), 1× phosphatase inhibitor cocktail 3 (Sigma-Aldrich), and 1× complete protease inhibitor cocktail (Roche Applied Science). Nuclei and cell debris were removed by centrifugation at 20,000*g* for 5 min at 4 °C. The protein concentrations of the supernatants were determined using the Pierce Protein Assay Kit (Thermo Scientific). Equal amounts of protein were separated using SDS-PAGE (M42010, GenScript) and transferred to a PVDF membrane (162-0177, Bio-Rad). Membranes were blocked in 5% milk in PBS for 1 h at RT and then incubated overnight at 4 °C with primary antibodies followed by incubation with HRP-conjugated secondary antibodies for 1 h at RT. Immunoreactive bands were visualized by chemiluminescence using Pierce Enhanced Chemiluminescent Western blotting Substrate (32106, Life Technologies), digitized, and then quantified using ImageJ software.

### Messenger RNA analysis

Levels of ATP-binding cassettes-A1, ATP-binding cassettes-G1, liver X receptor-α, monocyte chemoattractant protein-1, and macrophage inflammatory protein 1-α mRNA in macrophages were analyzed by quantitative RT-PCR. The macrophages were dissolved in TRIzol reagent (Invitrogen) for RNA extraction using Direct-zol RNA Miniprep reagents (Zymo Research). The RNA was reversed-transcribed using qScript cDNA synthesis kit (Quantabio). Quantitative real-time PCR was performed on a StepOnePlus Fast Thermocycler using Fast SYBR Green Master Mix (Applied Biosystems) with primers as indicated in Table S2. The levels of target mRNA were normalized to cyclophilin mRNA levels.

### Statistics

All data were expressed as the mean ± SD. Statistical analysis was performed using SigmaPlot, version 14.0, software (SYSTAT Software). Normality was examined using the Shapiro–Wilk test. Data with equal variance based on Levene’s analysis were then subjected to multiple group comparisons by one-way ANOVA with the Student–Newman–Keuls test or Holm–Sidak post hoc analysis. Student *t* test was used when evaluating differences between two groups. Differences at *p* < 0.05 were considered statistically significant.

## Data availability

The data supporting this study are available in the article and from the corresponding author (David Y. Hui) (huidy@ucmail.uc.edu) upon request.

## Supporting information

This article contains supporting information.

## Conflict of interest

The authors declare that they have no conflicts of interest with the contents of this article.

## References

[1] Dolgin E. (2017). The most popular genes in the human genome. Nature.

[2] Song Y., Stampfer M.J., Liu S. (2004). Meta-analysis: Apolipoprotein E genotypes and risk for coronary heart disease. Ann. Intern. Med..

[3] Couderc R., Mahieux F., Bailleul S., Fenelon G., Mary R., Fermanian J. (1993). Prevalence of apolipoprotein E phenotypes in ischemic cerebrovascular disease. A case-control study. Stroke.

[4] Senti M., Nogues X., Pedro-Botet J., Rubies-Prat J., Vidal-Barraquer F. (1992). Lipoprotein profile in men with peripheral vascular disease. Role of intermediate density lipoproteins and apoprotein E phenotypes. Circulation.

[5] de Andrade M., Thandi I., Brown S., Gotto A., Patsch W., Boerwinkle E. (1995). Relationship of the apolipoprotein E polymorphism with carotid artery atherosclerosis. Am. J. Hum. Genet..

[6] Davignon J., Gregg R.E., Sing C.F. (1988). Apolipoprotein E polymorphism and atherosclerosis. Atherosclerosis.

[7] Wilson P.W.F., Schaefer E.J., Larson M.G., Ordovas J.M. (1996). Apolipoprotein E alleles and risk of coronary disease. A meta analysis. Arterioscler. Thromb. Vasc. Biol..

[8] McCarron M.O., Delong D., Alberts M.J. (1999). APOE genotype as a risk factor for ischemic cerebrovascular disease: A meta-analysis. Neurology.

[9] Bennet A.M., Di Angelantonio E., Ye Z., Wensley F., Dahlin A., Ahlbom A., Keavney B., Collins R., Wiman B., de Faire U., Danesh J. (2007). Association of apolipoprotein E genotypes with lipid levels and coronary risk. JAMA.

[10] Anthopoulos P.G., Hamodrakas S.J., Bagos P.G. (2010). Apolipoprotein E polymorphisms and type 2 diabetes: A meta-analysis of 30 studies including 5423 cases and 8192 controls. Mol. Genet. Metab..

[11] Sima A., Iordan A., Stancu C. (2007). Apolipoprotein E polymorphism - a risk factor for metabolic syndrome. Clin. Chem. Lab. Med..

[12] Vaisi-Raygani A., Rahimi Z., Nomani H., Tavilani H., Pourmotabbed T. (2007). The presence of apolipoprotein epsilon4 and epsilon2 alleles augments the risk of coronary artery disease in type 2 diabetic patients. Clin. Biochem..

[13] Ramus S.M., Petrovic D. (2019). Genetic variations and subclinical markers of carotid atherosclerosis in patients with type 2 diabetes mellitus. Curr. Vasc. Pharmacol..

[14] Santos-Ferreira C., Baptista R., Oliveira-Santos M., Costa R., Moura J.P., Goncalves L. (2019). Apolipoprotein E2 genotype is associated with a 2-fold increase in the incidence of type 2 diabetes mellitus: Results from a long-term observational study. J. Lipids.

[15] Zeljko H., Skaric-Juric T., Narancic N., Tomas Z., Baresic A., Salihovic M., Starcevic B., Janicijevic B. (2011). E2 allele of the apolipoprotein E gene polymorphism is predictive for obesity status in Roma minority population of Croatia. Lipids Health Dis..

[16] Strittmatter W.J., Roses A.D. (1996). Apolipoprotein E and Alzheimer's disease. Annu. Rev. Neurosci..

[17] Mahley R.W., Weisgraber K.H., Huang Y. (2009). Apolipoprotein E: Structure determines function, from atherosclerosis to Alzheimer's disease to AIDS. J. Lipid Res..

[18] Getz G.S., Reardon C.A. (2009). Apoprotein E as a lipid transport and signaling protein in the blood, liver, and artery wall. J. Lipid Res..

[19] Lahoz C., Schaefer E.J., Cupples L.A., Wilson P.W.F., Levy D., Osgood D., Parpos S., Pedro-Botet J., Daly J.A., Ordovas J.M. (2001). Apolipoprotein E genotype and cardiovascular disease in the Framingham Heart Study. Atherosclerosis.

[20] Eto M., Watanabe K., Ishii K. (1986). Reciprocal effects of apolipoprotein E alleles (epsilon 2 and epsilon 4) on plasma lipid levels in normolipidemic subjects. Clin. Genet..

[21] Duman B.S., Ozturk M., Yilmazer S., Hatemi H. (2004). Apolipoprotein E polymorphism in Turkish subjects with type 2 diabetes mellitus: Allele frequency and relationship to serum lipid concentrations. Diabetes Nutr. Metab..

[22] Kalix B., Meynet M.C., Garin M.C., James R.W. (2001). The apolipoprotein epsilon2 allele and the severity of coronary artery disease in type 2 diabetic patients. Diabet. Med..

[23] Yamazaki Y., Zhao N., Caulfield T.R., Liu C.-C., Bu G. (2019). Apolipoprotein E and Alzheimer disease: Pathobiology and targeting strategies. Nat. Rev. Neurol..

[24] Mahley R.W., Rall S.C. (2000). Apolipoprotein E: Far more than a lipid transport protein. Annu. Rev. Genomics Hum. Genet..

[25] Pendse A.A., Arbones-Mainar J.M., Johnson L.A., Altenburg M.K., Maeda N. (2009). Apolipoprotein E knock-out and knock-in mice: Atherosclerosis, metabolic syndrome, and beyond. J. Lipid Res..

[26] Mahley R.W. (1988). Apolipoprotein E: Cholesterol transport protein with expanding role in cell biology. Science.

[27] Ishibashi S., Herz J., Maeda N., Goldstein J.L., Brown M.S. (1994). The two receptor model of lipoprotein clearance: Tests of the hypothesis in knockout mice lacking the low density lipoprotein receptor, apolipoprotein E, or both proteins. Proc. Natl. Acad. Sci. U. S. A..

[28] Rohlmann A., Gotthardt M., Hammer R.E., Herz J. (1998). Inducible inactivation of hepatic LRP gene by Cre-mediated recombination confirms role of LRP in clearance of chylomicron remnants. J. Clin. Invest..

[29] Yue L., Bian J.-T., Grizelj I., Cavka A., Phillips S.A., Makino A., Mazzone T. (2012). ApoE enhances endothelial-NO production by modulating caveolin-1 interaction with eNOS. Hypertension.

[30] Ulrich V., Konaniah E.S., Herz J., Gerard R.D., Jung E., Yuhanna I.S., Ahmed M., Hui D.Y., Mineo C., Shaul P.W. (2014). Genetic variants of apoE and apoER2 differentially modulate endothelial function. Proc. Natl. Acad. Sci. U. S. A..

[31] Ishigami M., Swertfeger D.K., Granholm N.A., Hui D.Y. (1998). Apolipoprotein E inhibits platelet-derived growth factor-induced vascular smooth muscle cell migration and proliferation by suppressing signal transduction and preventing cell entry to G1 phase. J. Biol. Chem..

[32] Baitsch D., Bock H.H., Engel T., Telgmann R., Muller-Tidow C., Varga G., Bot M., Herz J., Robenek H., von Eckardstein A., Nofer J.-R. (2011). Apolipoprotein E induces antiinflammatory phenotype in macrophages. Arterioscler. Thromb. Vasc. Biol..

[33] Schneider W.J., Kovanen P.T., Brown M.S., Goldstein J., Utermann G., Weber W., Havel R.J., Kotite L., Kane J., Innerarity T.L., Mahley R.W. (1981). Abnormal binding of mutant apoprotein E to low density lipoprotein receptors of human fibroblasts and membranes from liver and adrenal of rats, rabbits, and cows. J. Clin. Invest..

[34] Hui D.Y., Innerarity T.L., Mahley R.W. (1984). Defective hepatic lipoprotein receptor binding of beta-very low density lipoproteins from type III hyperlipoproteinemic patients. Importance of apolipoprotein E. J. Biol. Chem..

[35] Mahley R.W., Rall S.C., Scriver C.R., Beaudet A.L., Valle D., Sly W.S. (2001). The Metabolic and Molecular Basis of Inherited Disease.

[36] Austin S.A., Katusic Z.S. (2016). Loss of endothelial nitric oxide synthease promotes p25 generation and tau phosphorylation in a murine model of Alzheimer's disease. Circ. Res..

[37] Zanotti I., Pedrelli M., Poti F., Stomeo G., Gomaraschi M., Calabresi L., Bernini F. (2011). Macrophage, but not systemic, apolipoprotein E is necessary for macrophage reverse cholesterol transport *in vivo*. Arterioscler. Thromb. Vasc. Biol..

[38] Murphy A.J., Akhtari M., Tolani S., Pagler T.A., Bijl N., Kuo C.-L., Wang M., Sanson M., Abramowicz S., Welch C., Bochem A.E., Kuivenhoven J.A., Yvan-Charvet L., Tall A.R. (2011). ApoE regulates hematopoietic stem cell proliferation, monocytosis, and monocyte accumulation in atherosclerotic lesions in mice. J. Clin. Invest..

[39] Linton M.F., Atkinson J.B., Fazio S. (1995). Prevention of atherosclerosis in apolipoprotein E-deficient mice by bone marrow transplantation. Science.

[40] Bellosta S., Mahley R.W., Sanan D.A., Murata J., Newland D.L., Taylor J.M., Pitas R.E. (1995). Macrophage-specific expression of human apolipoprotein E reduces atherosclerosis in hypercholesterolemic apolipoprotein E-null mice. J. Clin. Invest..

[41] Van Eck M., Herijgers N., Van Dijk K.W., Havekes L.M., Hofker M.H., Groot P.H.E., Van Berkel T.J.C. (2000). Effect of macrophage-derived mouse apoE, human apoE3-leiden, and human apoE2 (arg158-cys) on cholesterol levels and atherosclerosis in apoE-deficient mice. Arterioscler. Thromb. Vasc. Biol..

[42] Fazio S., Babaev V.R., Burleigh M.E., Major A.S., Hasty A.H., Linton M.F. (2002). Physiological expression of macrophage apoE in the artery wall reduces atherosclerosis in severely hyperlipidemic mice. J. Lipid Res..

[43] van Eck M., Herijgers N., Vidgeon-Hart M., Pearce N.J., Hoogerbrugge P.M., Groot P.H.E., Van Berkel T.J.C. (2000). Accelerated atherosclerosis in C57BL/6 mice transplanted with apoE-deficient bone marrow. Atherosclerosis.

[44] Tsoi L.-M., Wong K.-Y., Liu Y.-M., Ho Y.-Y. (2007). Apoprotein E isoform-dependent expression and secretion of pro-inflammatory cytokines TNF-a and IL-6 in macrophages. Arch. Biochem. Biophys..

[45] Blazejewska-Hyzorek B., Gromadzka G., Skowronska M., Czlonkowska A. (2014). APOE e2 allele is an independent risk factor for vulnerable carotid plaque in ischemic stroke patients. Neurol. Res..

[46] Damsgaard C.T., Lauritzen L., Calder P.C., Kjaer T.M.R., Frokiaer H. (2009). Whole blood culture is a valid low-cost method to measure monocytic cytokines - a comparison of cytokine production in cultures of human whole blood, mononuclear cells and monocytes. J. Immunol. Methods.

[47] Li K., Ching D., Luk F.S., Raffai R.L. (2015). Apolipoprotein E enhances microRNA-146a in monocytes and macrophages to suppress nuclear factor-kB-driven inflammation and atherosclerosis. Circ. Res..

[48] Dinarello C.A. (1988). Interleukin-1. Ann. N. Y. Acad. Sci..

[49] Nagareddy P.R., Kraakman M., Masters S.L., Stirzaker R.A., Gorman D.J., Grant R.W., Dragoljevic D., Hong E.S., Abdel-Latif A., Smytrh S.S., Choi S.H., Korner J., Bornfeldt K.E., Fisher E.A., Dixit V.D. (2014). Adipose tissue macrophages promote myelopoiesis and monocytosis in obesity. Cell Metab..

[50] Lenkiewicz A.M., Adamiak M., Thapa A., Bujko K., Pedziwiatr D., Abdel-Latif A.K., Kucia M., Ratajczak J., Ratajczak M.Z. (2019). The NLRP3 inflammasom orchestrates mobilization of bone marrow-residing stem cells into peripheral blood. Stem Cell Rev. Rep..

[51] Chawla A., Boisvert W.A., Lee C.H., Laffitte B.A., Barak Y., B J.S., Liao D., Nagy L., Edwards P.A., Curtiss L.K., Evans R.M., Tontonoz P. (2001). A PPARgamma-LXR-ABCA1 pathway in macrophages is involved in cholesterol efflux and atherogenesis. Mol. Cell.

[52] Fan D., Qiu S., Overton C.D., Yancey P.G., Swift L.L., Jerome W.G., Linton M.F., Fazio S. (2007). Impaired secretion of apolipoprotein E2 from macrophages. J. Biol. Chem..

[53] Gordon V., Innerarity T.L., Mahley R.W. (1983). Formation of cholesterol- and apoprotein E-enriched high density lipoproteins *in vitro*. J. Biol. Chem..

[54] de la Haba C., Palacio J.R., Martinez P., Morros A. (2013). Effect of oxidative stress on plasma membrane fluidity of THP-1 induced macrophages. Biochim. Biophys. Acta.

[55] Powers K.A., Szaszi K., Khadaroo R.G., Tawadros P.S., Marshall J.C., Kapus A., Rotstein O.D. (2006). Oxidative stress generated by hemorrhagic shock recruits toll-like receptor 4 to the plasma membrane in macrophages. J. Exp. Med..

[56] Jofre-Monseny L., Minihane A.-M., Rimbach G. (2008). Impact of apoE genotype on oxidative stress, inflammation and disease risk. Mol. Nutr. Food Res..

[57] Groeneweg M., Kanters E., Vergouwe M.N., Duerink H., Kraal G., Hofker M.H., de Winther M.P.J. (2006). Lipolysaccharide-indued gene expression in murine macrophages is enhanced by prior exposure to oxLDL. J. Lipid Res..

[58] Fessler M.B., Parks J.S. (2011). Intracellular lipid flux and membrane microdomains as organizing principles in inflammatory cell signaling. J. Immunol..

[59] Bonacina F., Coe D., Wang G., Longhi M.P., Baragetti A., Moregola A., Garlaschelli K., Uboldi P., Pellegatta F., Grigore L., Da Dalt L., Annoni A., Gregori S., Xiao Q., Caruso D. (2018). Myeloid apolipoprotein E controls dendritic cell antigen presentation and T cell activation. Nat. Commun..

[60] Allan L.L., Hoefl K., Zheng D.-J., Chung B.K., Kozak F.K., Tan R., van den Elzen P. (2009). Apolipoprotein-mediated lipid antigen presentation in B cells provides a pathway for innate help by NKT cells. Blood.

[61] Major A.S., Joyce S., Van Kaer L. (2006). Lipid metabolism, atherogenesis and CD1-restricted antigen presentation. Trends Mol. Med..

[62] van den Elzen P., Garg S., Leon L., Brigl M., Leadbetter E.A., Gumperz J.E., Dascher C.C., Cheng T.-Y., Sacks F.M., Illarionov P.A., Besra G.S., Kent S.C., Moody D.B., Brenner M.B. (2005). Apolipoprotein-mediated pathways of lipid antigen presentation. Nature.

[63] Takahashi A., Hanson M.G.V., Norell H.R., Havelka A.M., Kono K., Malmberg K.-J., Kiessling R.V.R. (2005). Preference cell death of CD8^+^ effector memory (CCR7^-^CD45RA^-^) T cells by hydrogen peroxide-induced oxidative stress. J. Immunol..

[64] Gupta S., Young T., Yel L., Su H., Gollapudi S. (2007). Differential sensitivity of naive and subsets of memory CD4+ and CD8+ T cells to hydrogen peroxide-induced apoptosis. Genes Immunol..

[65] Ley K., Miller Y.I., Hedrick C.C. (2011). Monocyte and macrophage dynamics during atherogenesis. Arterioscler. Thromb. Vasc. Biol..

[66] Ammirati E., Cianflone D., Vecchio V., Banfi M., Vermi A.C., De Metrio M., Grigore L., Pellegatta F., Pirillo A., Garlaschelli K., Manfredi A.A., Catapano A.L., Maseri A., Palini A.G., Norata G.D. (2012). Effector memory T cells are associated with atherosclerosis in humans and animal models. J. Am. Heart Assoc..

[67] Olson N.C., Doyle M.F., Jenny N.S., Huber S.A., Psaty B.M., Kronmal R.A., Tracy R.P. (2013). Decreased naive and increased memory CD4^+^ T cells are associated with subclinical atherosclerosis: The multi-ethnic study of atherosclerosis. PLoS One.

[68] Cochain C., Koch M., Chaudhari S.M., Busch M., Pelisek J., Boon L., Zernecke A. (2015). CD8^+^ T cells regulate monopoiesis and circulating Ly6C^high^ monocyte levels in atherosclerosis in mice. Circ. Res..

[69] Cochain C., Zernecke A. (2016). Protective and pathogenic roles of CD8^+^ T cells in atherosclerosis. Basic Res. Cardiol..

[70] Kuhel D.G., Konaniah E.S., Basford J.E., McVey C., Goodin C.T., Chatterjee T.K., Weintraub N.L., Hui D.Y. (2013). Apolipoprotein E2 accentuates postprandial inflammation and diet-induced obesity to promote hyperinsulinemia in mice. Diabetes.

[71] Thomson L. (2015). 3-nitrotyrosine modified proteins in atherosclerosis. Dis. Markers.

[72] Murphy A.J., Dragoljevic D., Tall A.R. (2014). Cholesterol efflux pathways regulate myelopoiesis: A potential link to altered macrophage function in atherosclerosis. Front. Immunol..

[73] Swirski F.K., Libby P., Aikawa E., Alcaide P., Luscinskas F.W., Weissleder R., Pittet M.J. (2007). Ly-6C^hi^ monocytes dominate hypercholesterolemia-associated monocytosis and give rise to macrophages in atheromata. J. Clin. Invest..

[74] Tolani S., Pagler T.A., Murphy A.J., Bochem A.E., Abramowicz S., Welch C., Nagareddy P.R., Holleran S., Hovingh G.K., Kuivenhoven J.A., Tall A.R. (2013). Hypercholesterolemia and reduced HDL-C promote hematopoietic stem cell proliferation and monocytosis: Studies in mice and FH children. Atherosclerosis.

[75] Dang E.V., Cyster J.G. (2019). Loss of sterol metabolic homeostasis triggers inflammasomes - how and why. Curr. Opin. Immunol..

[76] Rajamaki K., Lappalainen J., Oorni K., Valimaki E., Matikainen S., Kovanen P.T., Eklund K.K. (2010). Cholesterol crystals activate the NLRP3 inflammasome in human macrophages: A novel link between cholesterol metabolism and inflammation. PLoS One.

[77] Westerterp M., Fotakis P., Ouimet M., Bochem A.E., Zhang H., Molusky M.M., Wang W., Abramowicz S., la Bastide-van Gemert S., Wang N., Welch C.L., Reilly M.P., Stroes E.S., Moore K.J., Tall A.R. (2018). Cholesterol efflux pathways suppress inflammasome activation, NETosis, and atherogenesis. Circulation.

[78] Maezawa I., Nivison M., Montine K.S., Maeda N., Montine T.J. (2006). Neurotoxicity from innate immune response is greatest with targeted replacement of E4 allele of apolipoproein E gene and is mediated by microglial p38MAPK. FASEB J..

[79] Maezawa I., Maeda N., Montine T.J., Montine K.S. (2006). Apolipoprotein E-specific innate immune response in astrocytes from targeted replacement mice. J. Neuroinflammation.

[80] Huebbe P., Lodge J.K., Rimbach G. (2010). Implications of apolipoprotein E genotype on inflammation and vitamin E status. Mol. Nutr. Food Res..

[81] Kawai T., Akira S. (2007). Signaling to NF-kB by toll-like receptors. Trends Mol. Med..

[82] Huang Y.-W.A., Zhou B., Nabet A.M., Wernig M., Sudhof T.C. (2019). Differential signaling mediated by apoE2, apoE3, and apoE4 in human neurons parallels Alzheimer's disease risk. J. Neurosci..

[83] Sullivan P.M., Mezdour H., Aratani Y., Knouff C., Najib J., Reddick R.L., Quarfordt S.H., Maeda N. (1997). Targeted replacement of the mouse apolipoprotein E gene with the common human apoE3 allele enhances diet-induced hypercholesterolemia and atherosclerosis. J. Biol. Chem..

[84] Arbones-Mainar J.M., Johnson L.A., Altenburg M.K., Maeda N. (2008). Differential modulation of diet-induced obesity and adipocyte functionality by human apolipoprotein E3 and E4 in mice. Int. J. Obes..

[85] Cash J.G., Kuhel D.G., Basford J.E., Jaeschke A., Chatterjee T.K., Weintraub N.L., Hui D.Y. (2012). Apolipoprotein E4 impairs macrophage efferocytosis and potentiates apoptosis by accelerating endoplasmic reticulum stress. J. Biol. Chem..

[86] Lee J.-M., Govindarajah V., Goddard B., Hinge A., Muench D.E., Filippi M.-D., Aronow B.J., Cancelas J.A., Salomonis N., Grimes H.L., Reynaud D. (2017). Obesity alters the long-term fitness of the hematopoietic stem cell compartment through modulation of Gfi1 expression. J. Exp. Med..

[87] Havel R.J., Eder H.A., Bragdon J.H. (1955). The distribution and chemical composition of ultracentrifugally separated lipoproteins in human serum. J. Clin. Invest..

[88] Waltmann M.D., Basford J.E., Konaniah E.S., Weintraub N.L., Hui D.Y. (2014). Apolipoprotein E receptor-2 deficiency enhances macrophage susceptibility to lipid accumulation and cell death to augment atherosclerotic plaque progression and necrosis. Biochim. Biophys. Acta.

