# Supplementary material for: Distinct pro-inflammatory properties of myeloid cell–derived apolipoprotein E2 and E4 in atherosclerosis promotion
Source: J Biol Chem. 2021 Aug 21;297(3):101106. doi: 10.1016/j.jbc.2021.101106 (PMC8437825; doi:10.1016/j.jbc.2021.101106)

SUPPORTING INFORMATION

Table S1. Antibodies used

Target antigen	Vendor or Source	Catalog #	Working concentration	Persistent ID / URL
NLRP3	Abcam	Ab263899	1:1000	RRID: AB_2889890
Caspase 1	Abcam	EPR16883	1:1000	RRID: AB_2884954
β-actin	Cell Signaling	4970L	1:1000	RRID: AB_2223172
GAPDH	Cell Signaling	2118S	1:1000	RRID: AB_561053
Sca1	Life Technologies	45-5981-82	1:100	RRID: AB_914732
c-kit	Life Technologies	17-1171-82	1:100	RRID: AB_469430
CD48	Life Technologies	47-0481-82	1:100	RRID: AB_2573962
CD34 (FITC)	eBioscience	11-0341-82	1:100	RRID: AB_465021
CD49	Life Technologies	12-5971-83	1:100	RRID: AB_466074
CD16/CD32	Life Technologies	25-0161-82	1:100	RRID: AB_469598
CD11b	BD Biosciences	557657	1:100	RRID: AB_396772
CD115	eBioscience	12-1152-82	1:100	RRID: AB_465808
Ly6G	BD Biosciences	560602	1:100	RRID: AB_1727563
Ly6C	BD Biosciences	560595	1:100	RRID: AB_1727554
CD3e (PerCP-Cy5.5)	BD Biosciences	551163	2 μg/ml	RRID: AB_394082
CD4 (FITC)	Invitrogen	11-0042-82	2.5 μg/ml	RRID: AB_464896
CD8a (APC-Cy7)	Life Technologies	A15386	10 μg/ml	RRID: AB_2534400
CD44	BioLegend	103012	2.5 μg/ml	RRID: AB_312963
CD62L (PE-Cy7)	BioLegend	104418	2.5 μg/ml	RRID: AB_313103
IL-1β	Novus	NB600-633	1:200	RRID: AB_10001060
Nitrotyrosine	Invitrogen	A21285	1:200	RRID: AB_221457
Anti-rabbit IgG (HRP)	Cell Signaling	7074S	1:2000	RRID: AB_2099233

Table S2. Primers used

Name	Sequence
ABCA1 forward	ACCCACCCTACGAACAACATGAGT
ABCA1 reverse	AAAGTTTCCAACAACACCGGGAGC
ABCG1 forward	AAAGGTCTCCAATCTCGTGCCGTA
ABCG1 reverse	TATAGTCAGCGTCACACATGCCCT
MCP-1 forward	CTTCCTCCACCACCATGCA
MCP-1 reverse	CCAGCCGGCAACTGTGA
MIP-1α forward	TTTGAAACCAGCAGCCTTTGCTCC
MIP-1α reverse	TCAGGCATTCAGTTCCAGGTCAGT
PPARγ forward	CTGCAGGCCCTGGAACTG
PPARγ reverse	CGATCTGCCTGAGGTCTGTCA
LXRα forward	CGACAGAGCTTCGTCCACAA
LXRα reverse	GCTCGTTCCCCAGCATTTT
Cyclophilin forward	TCATGTGCCAGGGTGGTGAC
Cyclophilin reverse	CCATTCAGTCTTGGCAGTGC

Figure S1. Gating strategies used for flow cytometry analysis of T lymphocyte subsets

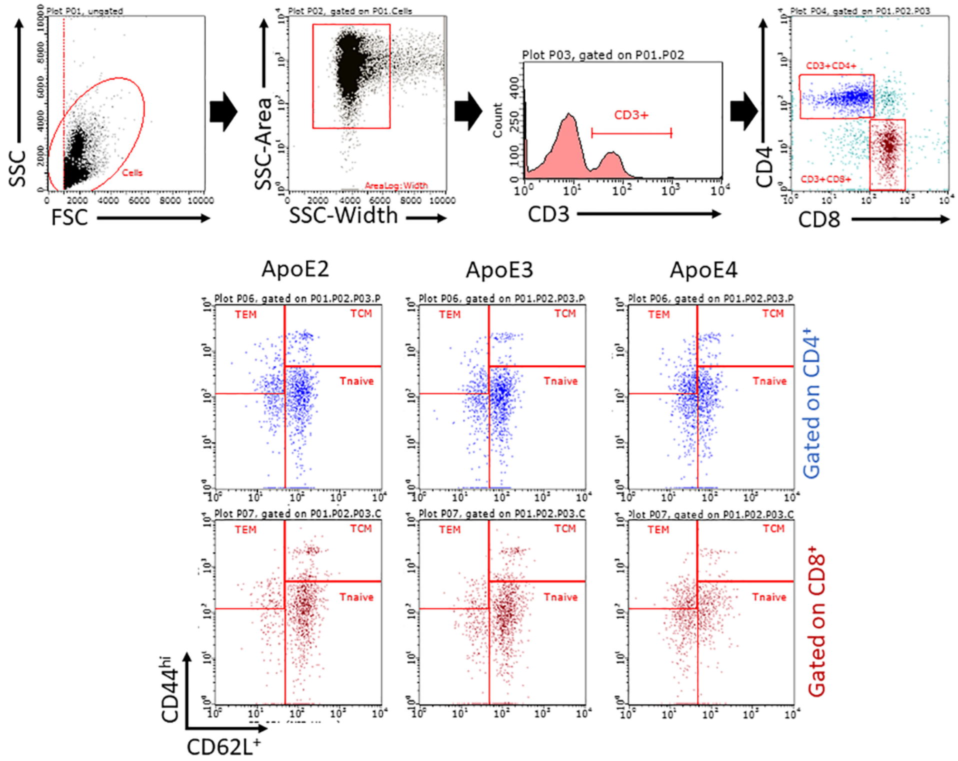

Supplement: Supplemental Tables S1, S2 and Figure S1 [file mmc1.pdf]
